# Minimal model of interictal and ictal discharges “Epileptor-2”

**DOI:** 10.1371/journal.pcbi.1006186

**Published:** 2018-05-31

**Authors:** Anton V. Chizhov, Artyom V. Zefirov, Dmitry V. Amakhin, Elena Yu. Smirnova, Aleksey V. Zaitsev

**Affiliations:** 1 Laboratory of Molecular Mechanisms of Neural Interactions, Sechenov Institute of Evolutionary Physiology and Biochemistry of the Russian Academy of Sciences, Saint Petersburg, Russia; 2 Computational Physics Laboratory, Ioffe Institute, Saint Petersburg, Russia; 3 Institute of Experimental Medicine, Almazov National Medical Research Centre, Saint Petersburg, Russia; University of California San Diego, UNITED STATES

## Abstract

Seizures occur in a recurrent manner with intermittent states of interictal and ictal discharges (IIDs and IDs). The transitions to and from IDs are determined by a set of processes, including synaptic interaction and ionic dynamics. Although mathematical models of separate types of epileptic discharges have been developed, modeling the transitions between states remains a challenge. A simple generic mathematical model of seizure dynamics (Epileptor) has recently been proposed by Jirsa et al. (2014); however, it is formulated in terms of abstract variables. In this paper, a minimal population-type model of IIDs and IDs is proposed that is as simple to use as the Epileptor, but the suggested model attributes physical meaning to the variables. The model is expressed in ordinary differential equations for extracellular potassium and intracellular sodium concentrations, membrane potential, and short-term synaptic depression variables. A quadratic integrate-and-fire model driven by the population input current is used to reproduce spike trains in a representative neuron. In simulations, potassium accumulation governs the transition from the silent state to the state of an ID. Each ID is composed of clustered IID-like events. The sodium accumulates during discharge and activates the sodium-potassium pump, which terminates the ID by restoring the potassium gradient and thus polarizing the neuronal membranes. The whole-cell and cell-attached recordings of a 4-AP-based in vitro model of epilepsy confirmed the primary model assumptions and predictions. The mathematical analysis revealed that the IID-like events are large-amplitude stochastic oscillations, which in the case of ID generation are controlled by slow oscillations of ionic concentrations. The IDs originate in the conditions of elevated potassium concentrations in a bath solution via a saddle-node-on-invariant-circle-like bifurcation for a non-smooth dynamical system. By providing a minimal biophysical description of ionic dynamics and network interactions, the model may serve as a hierarchical base from a simple to more complex modeling of seizures.

## Author summary

In pathological conditions of epilepsy, the functioning of the neural network crucially depends on the ionic concentrations inside and outside neurons. A number of factors that affect neuronal activity is large. That is why the development of a minimal model that reproduces typical seizures could structure further experimental and analytical studies of the pathological mechanisms. Here, on a base of known biophysical models, we present a simple population-type model that includes only four principal variables, the extracellular potassium concentration, the intracellular sodium concentration, the membrane potential and the synaptic resource diminishing due to short-term synaptic depression. A simple modeled neuron is used as an observer of the population activity. We validate the model assumptions with in vitro experiments. Our model reproduces ictal and interictal events, where the latter result in bursts of spikes in single neurons, and the former represent the cluster of spike bursts. Mathematical analysis reveals that the bursts are spontaneous large-amplitude oscillations, which may cluster after a saddle-node on invariant circle bifurcation in the pro-epileptic conditions. Our consideration has significant bearing in understanding pathological neuronal network dynamics.

## Introduction

A simple canonical mathematical model of epileptic discharges has been proposed by V. Jirsa et al. [1] based on an analysis of experimental recordings of epileptic discharges in humans and animal models. A comparison of the characteristic features of the canonical bifurcations of simple dynamical systems with the elements of the experimental signals revealed the most typical type of transitions to and from the ictal state. A model called Epileptor, which reproduces this behavior, has been proposed. The model can be expressed in a set of six ordinary differential equations [2]. This model led to a series of predictions and applications in physiology, neuroinformatics, and translational medicine [2–4]. A disadvantage of the Epileptor is a lack of physical meaning for its governing variables. A biophysical interpretation would help clarify the experimental validation of the model’s predictions. In the present work, an alternative model with a similar mathematical simplicity but that operates with physically meaningful variables is proposed. The model is referred to as the Epileptor-2. The set of variables chosen to describe the epileptic state transitions along with the interpretation of the terms included in the equations constitute the primary model predictions. Namely, the model distinguishes between main variables: two ionic concentrations of the extracellular potassium and intracellular sodium, a membrane potential, and a synaptic resource. The selection of these variables as well as the forms of the governing equations with experimental observations taken from both the experiments and the literature are justified and described in detail.

While formulating the model assumptions, special attention was focused on two specific experimental observations. First, in a number of experiments, IDs were formed from a clustered number of short discharges [5–8]. These elementary discharges are similar to IIDs. During each IID-like event, a principal neuron typically generated a burst of spikes. Thus, the ID represents a burst of bursts of spikes. Previous biophysical seizure-modeling attempts that considered the ionic dynamics in a single neuron model [9,10] or a network [11–13] did not show this feature. The second observation suggested a crucial role of the Na-K pump in the termination of each ID. In fact, the extracellular potassium concentration increased during the initial phase of the ID and began to decrease before the end of the ID [4,8,14], whereas the intracellular sodium concentration increased until the actual end of the ID [15]. Some simulations showed a similar behavior of ionic concentrations [9,10]. Therefore, the ionic dynamics may be explained by the activation of the Na-K pump, and the consequent decrease in the extracellular potassium concentration may lead to ID termination [12]. The two experimental observations described have not been modeled together. The Results section of the paper is organized as follows. First, the proposed model is presented, which is then justified. The simulations of the interictal and ictal discharges are described and compared with experimental data in order to validate the main assumptions of the model. Then, the main component of the Epileptor-2’s dynamics is discussed, which is an interictal-like discharge observed as a short burst of spikes. To study these discharges, the case of constant ionic conductance was considered, the Epileptor-2 model was reduced to a second-order system of stochastic differential equations, and its mathematical analysis was carried out. It is shown that the deterministic system of the reduced model has the only stable equilibrium as an attractor; however, under noise, the model exhibits a rather complex behavior of the stochastic generation of bursting, similar to the behavior of the stochastic Hindmarsh-Rose model [16]. For weak noise, random states concentrate near the equilibrium. With an increase of noise intensity, random trajectories can travel far from the stable equilibrium, and along with small-amplitude oscillations around the equilibrium, bursts are observed. These bursts constitute the interictal and ictal events in the simulations of Epileptor-2. The slow oscillations of the potassium and sodium ionic concentrations were then analyzed, which determine the origination and shape of seizures.

## Results

### Governing equations of the Epileptor-2

**Fig 1:**
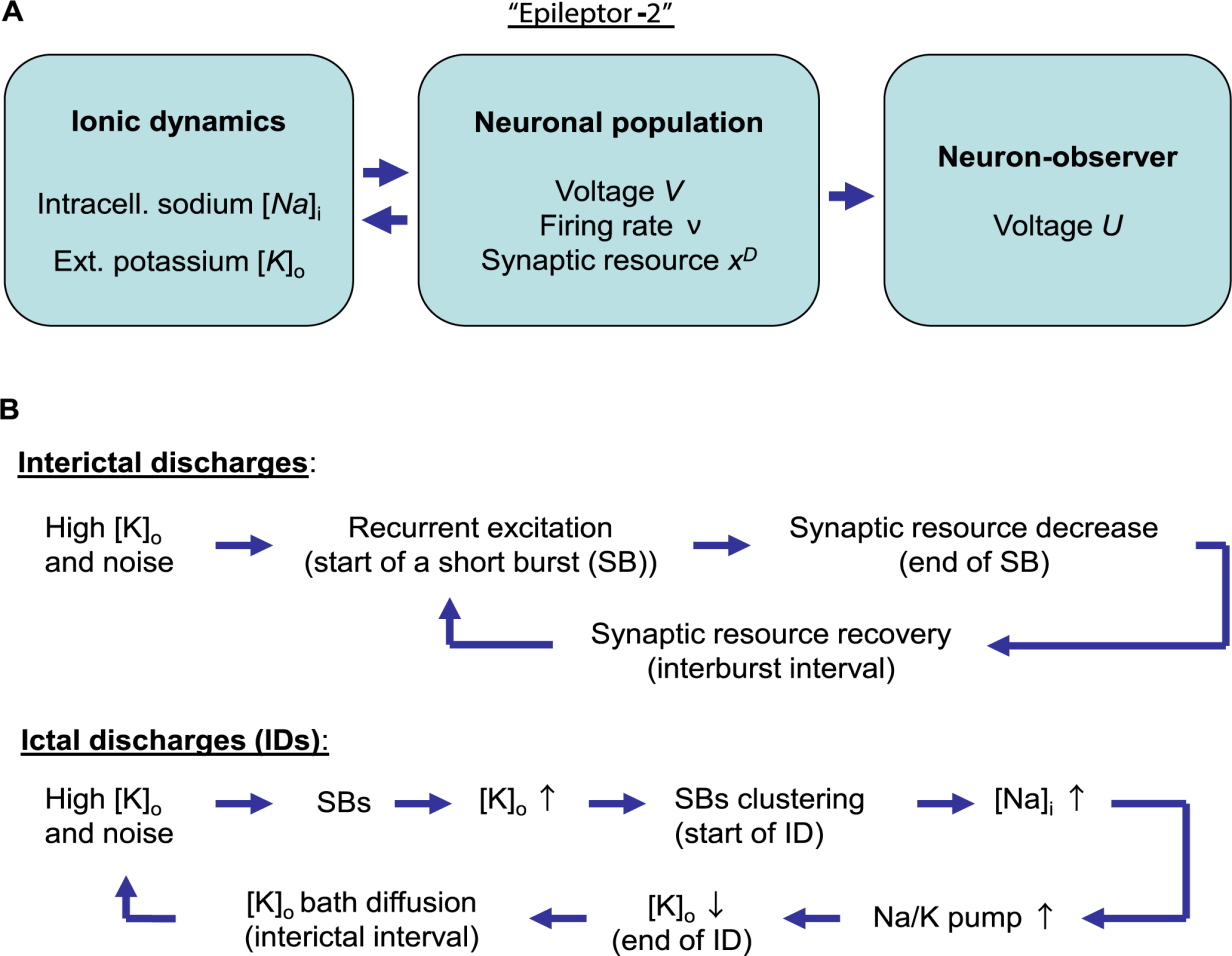
Schematic of Epileptor-2 and the described mechanisms of interictal and ictal discharges. (**A**) The model consists of the equations that describes the ionic dynamics, the neuronal population excitation and the activity of a single representative neuron driven by the population. (**B**) Mechanisms of the discharges, explained in the main text.

The proposed model consists of three subsystems that describe: (i) the ionic dynamics, (ii) the neuronal excitability, and (iii) a neuron-observer (Fig 1A).

The proposed population model consists of the following equations:

$$\frac{d{\left[K\right]}_{o}}{dt}=\frac{{\left[K\right]}_{bath}-{\left[K\right]}_{o}}{\tau_{K}}-2\gamma \;I_{pump}+\delta {\left[K\right]}_{o}\nu (t)$$ (1)

$$\frac{d{\left[Na\right]}_{i}}{dt}=\frac{{\left[Na\right]}_{i}^{0}-{\left[Na\right]}_{i}}{\tau_{Na}}-3\;I_{pump}+\delta {\left[Na\right]}_{i}\;\nu (t)$$ (2)

$$C\frac{dV}{dt}=-g_{L}\;V+u(t)$$ (3)

$$\frac{dx^{D}}{dt}=\frac{1-x^{D}}{\tau_{D}}-\delta x_{}^{D}\;x^{D}\;\nu (t)$$ (4)

All the terms of these equations are explained in the next section. Here, only the main variables are introduced, which are as follows: [*K*]_*o*_ and [*Na*]_*i*_ represent extracellular potassium and intraneuronal sodium concentrations, respectively; *V*(*t*) is the membrane depolarization; *x*^*D*^(*t*) is the synaptic resource; *ν*(*t*) is the firing rate of an excitatory population; and an inhibitory population firing rate is assumed to be proportional to *ν*(*t*). The dynamics described by these equations is driven *ν*(*t*), which is calculated with a sigmoidal input-output function:

$$\nu (V(t))=\nu_{\max}\;{\left[\frac{2}{1+\exp\left[-2\left(V(t)-V_{th}\right)/k_{\nu }\right]}-1\right]}_{+}$$ (5)

where [*x*]_+_ is equal to x for the positive argument and 0 otherwise. The input current *u*(*t*) includes the potassium depolarizing current, the synaptic drive, and the noise *ξ*(*t*), respectively:

$$u(t)=g_{K,\;leak}(V_{K}(t)-V_{K}^{0})+G_{syn}\;\nu (t)\;(x^{D}(t)-0.5)+\sigma \;\xi (t)$$ (6)

The potassium reversal potential is obtained from the ion concentrations via the Nernst equation:

$$V_{K}=26.6\;mV\;\;\ln\left({\left[K\right]}_{o}/130mM\right)$$ (7)

The Na^+^/K^+^ pump current is taken from Cressman et al. [9] in the form:

$$I_{pump}=\frac{\rho }{\left(1+\exp(3.5-{\left[K\right]}_{o}))(1+\exp((25-{\left[Na\right]}_{i})/3)\right)}$$ (8)

The basic set of parameters is as follows. The time constants for ionic dynamics, membrane polarization, and synaptic depression are *τ*_*K*_ = 100*s*, *τ*_*Na*_ = 20*s*, *τ*_*m*_ = *C*/*g*_*L*_ = 10*ms*, and *τ*_*D*_ = 2*s*; the concentration increments at spike are *δ*[*K*]_*o*_ = 0.02*mM*, *δ*[*Na*]_*i*_ = 0.03*mM*, *δx*^*D*^ = 0.01; the Gaussian white noise *ξ*(*t*) has zero mean and unity dispersion, ⟨*ξ*(*t*)*ξ*(*t*')⟩ = *τ*_*m*_ *δ*(*t*−*t*'); the noise amplitude is *σ*/*g*_*L*_ = 25*mV*; the maximum pump flux is *ρ* = 0.2*mM*/*s*; the volume ration is *γ* = 10 (close to 7 given in [17]); the postsynaptic charge is *G*_*syn*_/*g*_*L*_ = 5*mV* ∙ *s*; the potassium leak conductance is *g*_*K*,*leak*_/*g*_*L*_ = 0.5; the initial extracellular and elevated bath potassium concentrations are ${\left[K\right]}_{O}^{0}=3\;mM$ and [*K*]_*bath*_ = 8.5*mM*; $V_{K}^{0}=26.6\;mV\;\;\ln\left({\left[K\right]}_{O}^{0}/130\;mM\right)$; initial and resting intracellular sodium concentrations are the same, ${\left[Na\right]}_{i}^{0}=10\;mM$. The parameters of the input-output function are the maximal rate *ν*_max_ = 100*Hz*, the threshold potential *V*_*th*_ = 25*mV*, and the gain *k*_*ν*_ = 20*mV*.

A representative neuron was modeled with a quadratic integrate-and-fire neuron [18, 19]. The equations for the membrane potential *U*(*t*) are as follows:

$$C_{U}\frac{dU}{dt}=g_{U}(U-U_{1})(U-U_{2})+u$$ (9)

$$if\;U>V^{T}\;then\;U=V_{reset}$$ (10)

with the parameters: *g*_*U*_ = 0.4*nS*/*mV*, *C*_*U*_ = 200*pF*, *V*^*T*^ = 25*mV*, *V*_*reset*_ = −50*mV*, *U*_1_ = −60*mV*, *U*_2_ = −40*mV*; an initial condition is *U* = −70*mV*.

### Phenomenological derivation and justification of the model

**Eq 1.** The model states that the elevation of the extracellular potassium concentration [*K*]_*o*_ plays a primary role in self-regenerating IDs. Before each ID, the extracellular potassium concentration accumulates after a series of IIDs. Each IID involves the activation of interneurons [5,7], which results in opening GABAergic channels that are permeable for chloride and bicarbonate [20] and chloride flux into neurons (note that due to bicarbonate participation, the reversal potential of the GABAergic current (*V*_GABA_) is more positive than that for chloride; thus, even if the membrane voltage converges to *V*_GABA_, the chloride is still transported into the neurons). The increase in intracellular chloride activates the KCC2 cotransporter, which carries the potassium out of the cells [21]. Thus, the extracellular potassium concentration increases at each IID, which was proven experimentally (see Fig 12 from [5] for example).

Different types of IIDs were reported in the literature. Some types are based on pure recurrent GABAergic interactions between interneurons [7,22]. In this case, interneurons excite each other due to the depolarizing GABA effect; however, they evoke only subthreshold voltage fluctuations in the principal cells. Other types of IIDs indicate that the IIDs recruit both GABAergic and glutamatergic synaptic interactions [5]. In some cases, interneurons excite the glutamatergic principal neurons due to the depolarizing GABA effect, and then the principal neurons excite both populations [7]. In other cases, the glutamatergic neurons begin the discharges, and interneurons are involved afterward [23]. It was noted that in a rough consideration of the time scale of one-hundred milliseconds, the spiking activity of interneurons is proportional to that of pyramidal cells. It is denoted as *ν*(*t*) in the model equations. Thus, the chloride accumulation and subsequent potassium elevation by the potassium-chloride cotransporters is roughly proportional to *ν*(*t*), as expressed by the final term in Eq 1. Another contribution to the final term in Eq 1 is the potassium voltage-gated channels. The total current is also roughly proportional to the spiking activity. Also, glutamatergic currents contribute to the potassium flux [24]. Again, they are roughly proportional to the presynaptic firing rate and thus to *ν*(*t*). All these contributions are efficiently taken into account by the final term in Eq 1.

Between IIDs, the potassium concentration [*K*]_*o*_ tends to return to the bath concentration due to diffusion and glial buffering. These processes are expressed by the first term in the right-hand side (r.h.s) of Eq 1, and the characteristic time is *τ*_*K*_. The potassium is pumped into the neurons by the ATP-dependent Na-K pump. In Eq 1, this outflux affects the extracellular potassium concentration [*K*]_*o*_ through the second term in the r.h.s.

**Eq 2.** The intracellular sodium concentration [*Na*]_*i*_ increases due to the firing activity *ν*(*t*), as expressed by the final term in Eq 1. The same term implies a sodium flux through glutamatergic synapses [25], whose activation is roughly proportional to the firing rate *ν*(*t*). The sodium is pumped from the neurons by the ATP-dependent Na-K pump. In Eq 2, this outflux affects [*Na*]_*i*_ through the second term in the r.h.s. The leakage and intracellular diffusion are taken into account by the first term in the r.h.s. of Eq 2. The characteristic time of the joint effect is evaluated by *τ*_*Na*_.

**Eq 3.** Describes a mean membrane depolarization due to the input *u(t)* using a single-compartment leaky neuron model. Not taking into account a spike generation, the voltage *V*(*t*) reflects a nominal, extreme level of membrane polarization. Together with Eq 6, it is a stochastic ordinary differential equation, which corresponds to the Ornstein-Uhlenbeck process that provides colored Gaussian noise with the temporal correlation scale *τ*_*m*_ [26].

**Eq 4.** Describes the short-term synaptic depression according to the Tsodyks-Markram model [27] written in the rate-dependent form [28].

**Fig 2:**
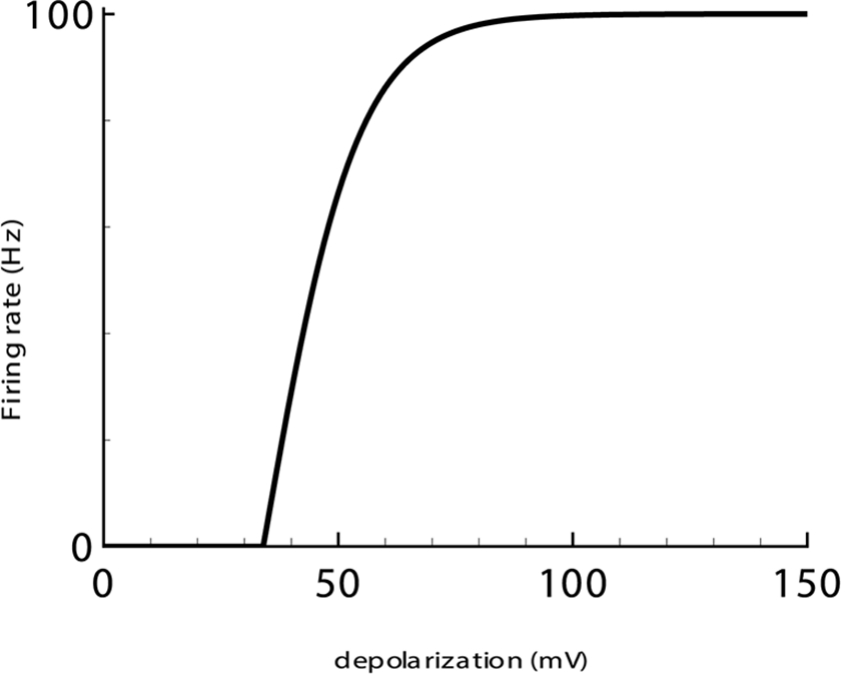
Firing rate-versus-depolarization dependence, Eq 5.

**Eq 5.** Describes the sigmoid-shaped dependence of the firing rate on depolarization *V(t)* (Fig 2). The dependence is not strictly valid for the transient states. Thus, it does not describe synchronization of neurons within a population and therefore not reproduce sharp peaks of the firing rate that are caused by this effect; however, it satisfactorily approximates activity averaged over a few tens of milliseconds [29]. A synaptic conductance is also missing in the relation. Thus its shunting effect is neglected. Thus, inhibition is taken into account implicitly as a negative term in the expression for *u(t)* (Eq 6).

**Eq 6.** For the neuronal input current *u(t)*, Eq 6 takes the depolarizing effect of the potassium concentration increase relative to its initial concentration (term 1), the total synaptic current (term 2), and the noise (term 3) into account. The current *u(t)* that determines the firing rate by Eq 5 does not contain intrinsic voltage-gated currents but reflects the indirect effects of the ionic concentrations on membrane polarization through the voltage-gated and leak channels. Term 1 is a linear approximation of the dependence of a sum of potassium channels on their reversal potential deflection $V_{K}(t)-V_{K}^{0}$. The potassium leak current provides the main contribution to this term. Thus, the coefficient of proportionality *g*_*K*,*leak*_ is called the potassium leak conductance. At the same time, the chloride leak current, which is normally smaller than the potassium leak current [30], also contributes to this term. This is in accordance with the previous assumption on the cooperative change of intracellular chloride and extracellular potassium concentrations. The change in the chloride leak current depends on the change in the chloride reversal potential, which is roughly proportional to $V_{K}(t)-V_{K}^{0}$, thus contributing to term 1.

In comparison with term 1, which evaluates the effects of potassium and chloride ions, the effect of the sodium concentration change on membrane polarization is small because the dependence of the resting potential on the sodium concentration (through the leak sodium current) is at least twice lower [30]. Therefore, the effect of sodium on *u(t)* was not considered.

The expression for the synaptic current (term 2) implies additional assumptions. It consists of two parts, a positive *G*_*syn*_ *ν*(*t*) *x*^*D*^(*t*) and negative −0.5 *G*_*syn*_ *ν*(*t*). The positive component of term 2 is an excitatory current; its approximation accounts for the synaptic resource *x*^*D*^ and its dependence on the firing rate *ν*(*t*), which implies that synaptic kinetics are instantaneous and assumes the driving force to be constant for the sake of simplicity. Hence, the coefficient *G*_*syn*_ is the maximum postsynaptic charge in response to a single presynaptic spike. The critical role of the short-term depression of excitatory synapses in the termination of IIDs has been revealed for glutamate-based [23] and depolarized GABA-based IIDs [31]. The role of short-term plasticity in inhibitory synapses does not seem to be essential. Thus, the negative component of term 2 is inhibition, which is non-depressing and proportional to the excitation with a coefficient 0.5. The value of the factor is roughly estimated to be less than 1 to provide self-sustaining recurrent excitation at the beginning of discharges when the synaptic resource is full. Below, the proportionality and the estimation of the factor are validated based on the experimental results.

The remainder of the equations, Eqs 7 and 8, are canonical [9]. Following the works by Bazhenov et al. [30] and Krishnan and Bazhenov [32], we assume that the sodium pump approximation elaborated for a single neuron [9] is valid for the description of the population level ion dynamics.

### Simulations

After applying the basic set of parameter values and manipulating one of the parameters of the Epileptor-2 model, two regimes of activity with IIDs and IDs were stimulated. As explained in this section, in both regimes, the discharges are composed of spontaneous short pulses that last a fraction of a second and correspond to short bursts (SBs) of spikes in single neurons.

**Fig 3:**
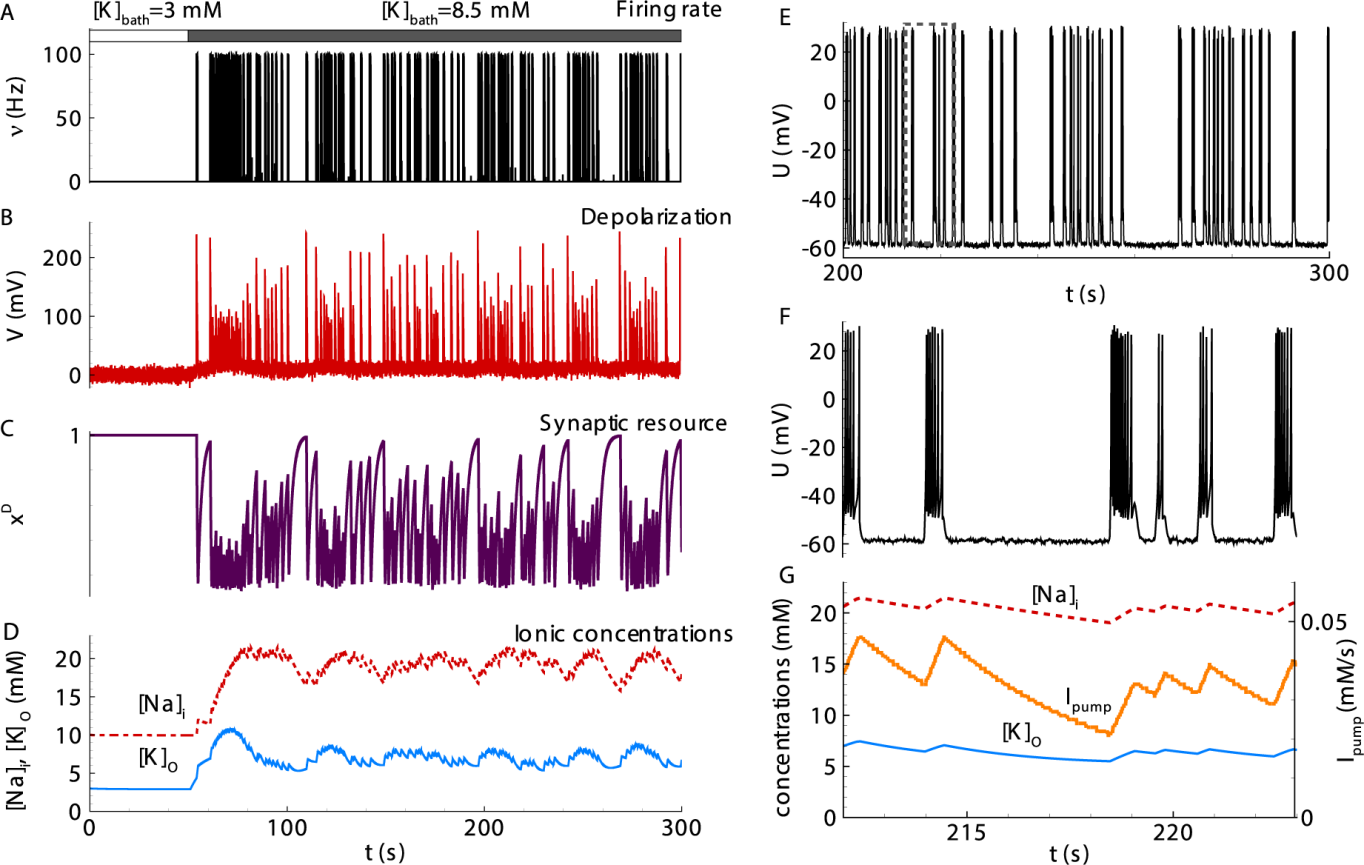
Simulation of an interictal regime with spontaneous short bursts (SBs). (**A**) The population firing rate. (**B**) The nominal depolarization. (**C**) The synaptic resource. (**D**) The intracellular sodium (red line) and extracellular potassium (blue) concentrations. (**E**) The membrane voltage in a representative neuron. (**F**) The membrane voltage on the time interval containing four SBs and marked by the dashed line in E. (**G**) The ionic flux through the Na-K-pump (orange, right axis) and the ionic concentrations (left axis) zoomed from D. Population variables in A-D and G were calculated with Eqs 1–8 with the basic parameter set except one different value, *τ*_*K*_ = 10*s*. The neuron membrane potential in E, F was obtained from Eqs 9 and 10. Note a lack of a strong clustering of SBs.

**Regime with interictal discharges.** In the case of a fast relaxation of the potassium concentration (the parameter *τ*_*K*_ = 10 *s* was different from the basic parameter values), the model presents a regime with spontaneous short bursts (SBs), which appear shortly after the onset of pro-epileptic bath solution with [*K*]_*bath*_ = 8.5*mM* (Fig 3). In a regime of non-clustered bursts, these SBs represent IIDs. The decreased *τ*_*K*_ may correspond, for example, to the case of a more efficient extracellular diffusion of potassium ions. This diffusion equilibrates the level of the potassium concentration between IIDs, thus preventing a cluster formation.

The mechanism of the IIDs is shown in Fig 1B. The increase of [*K*]_*o*_ from the initial level towards the bath concentration due to diffusion leads to a *u*(*t*) increase. The generation of SB begins when the input current *u*(*t*) leads the depolarization *V*(*t*) close to the threshold *V*_*th*_ *g*_*L*_. According to Eq 5, the firing rate increases and recurrently affects *u*(*t*) in accordance to Eq 6. The synaptic resource *x*^*D*^(*t*) begins to vanish, the current *u*(*t*) decreases, and SB terminates. During each SB, a representative neuron generates a few spikes (Fig 3F). The spike generation terminates due to the decrease of *u*(*t*).

The ionic concentrations [*K*]_*o*_ and [*Na*]_*i*_ increase at each SB and then relax during the interburst intervals (Fig 3G). The mean levels of the ion concentrations remain approximately constant. Note that the regime with only IIDs is obtained with relatively fast [*K*]_*o*_ relaxation (*τ*_*K*_ = 10*s*) compared with the characteristic interburst interval. This fast relaxation prevents any significant potassium accumulation during the train of SBs.

**Fig 4:**
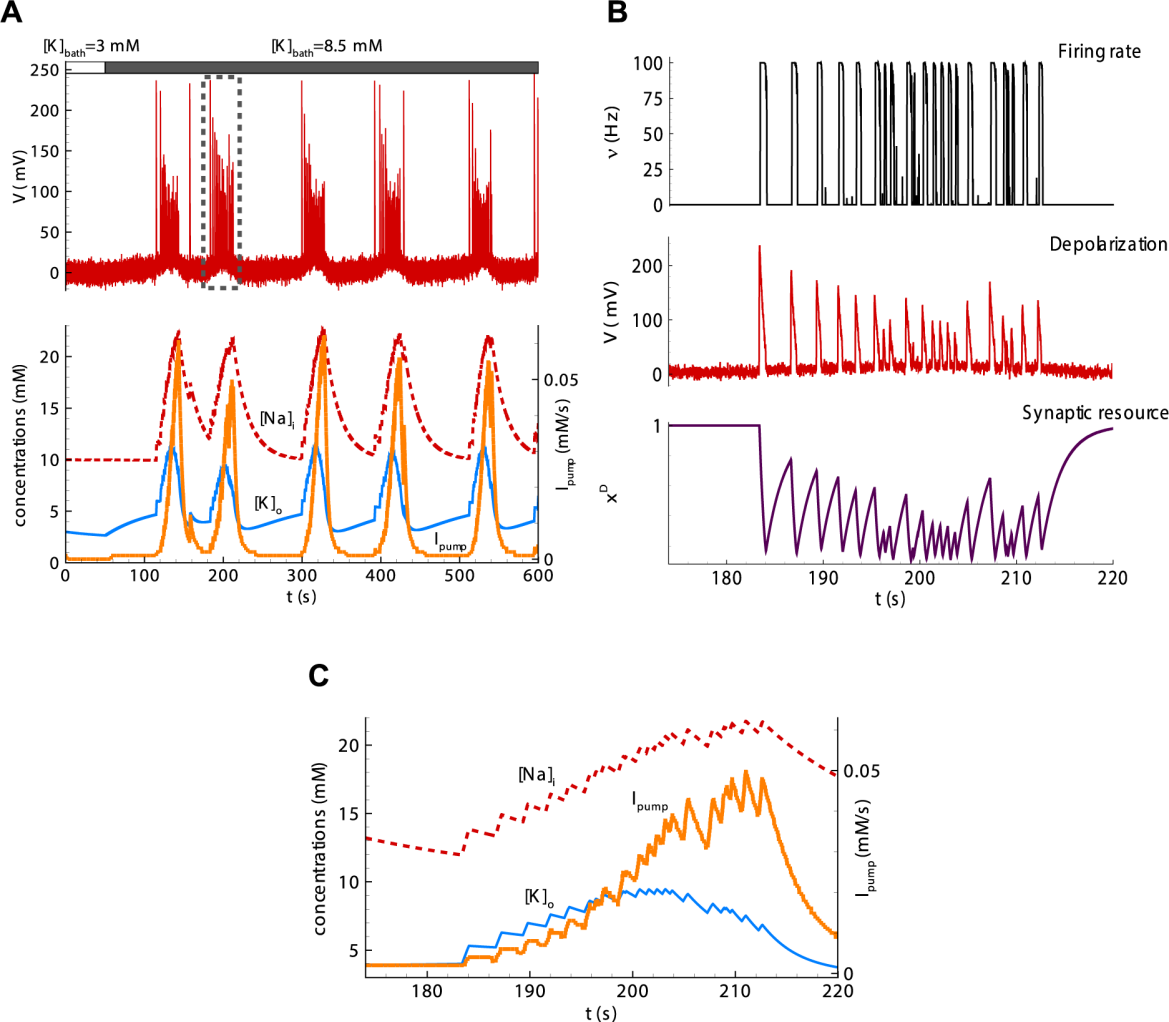
Simulations of IDs, each consisting of separate short bursts. (**A**) The nominal depolarization (top plot), the intracellular sodium (red line) and extracellular potassium (blue) concentrations (bottom, left axis), and the ionic flux through the Na-K-pump (orange line, bottom plot, right axis) during six IDs. (**B**) The population firing rate (black), the nominal depolarization (red), and the synaptic resource (violet) during a single ID consisting of some SBs. (**C**) Zoomed traces from A, bottom during a single ID. Simulations were done with Eqs 1–8 with the basic parameter set. Note the decrease of [*K*]_*o*_ at the high level of peaks of *I*_*pump*_ and following the termination of the ID.

**Fig 5:**
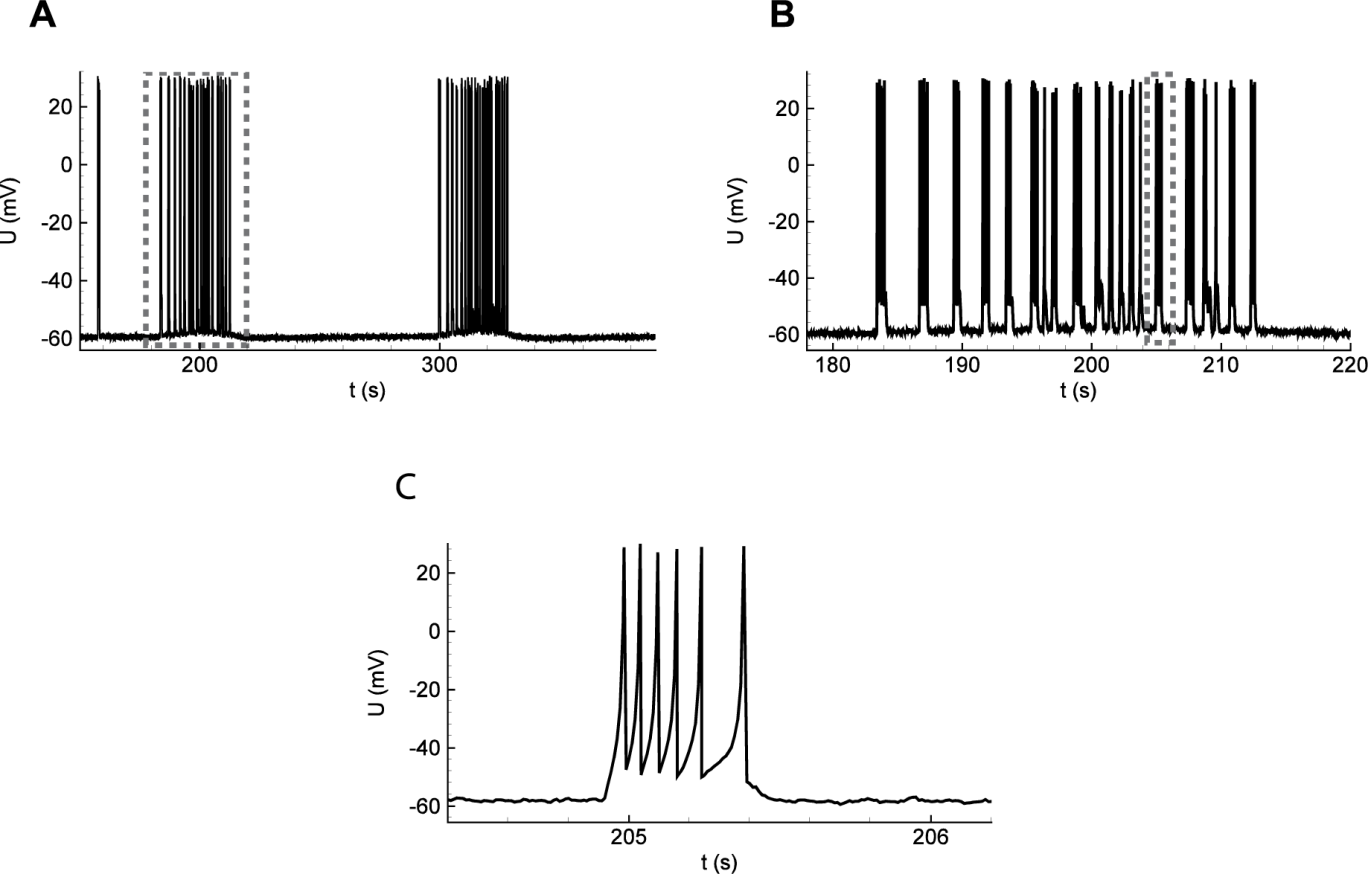
Simulations of a single neuron activity during the IDs shown in Fig 4. (**A**) Two IDs as bursts of clustered SBs, seen in the membrane voltage. (**B**) A single ID containing a number of SBs. Membrane voltage. (**C**) A single SB.

**Regime with ictal discharges.** The simulations of the model based on Eqs (1–8) with the basic parameter set show a population activity with recursive spontaneous IDs (Fig 4A), shortly after the onset of pro-epileptic bath solution with [*K*]_*bath*_ = 8.5*mM*. Each ID is characterized by a high rate of activity (black line) for 30 seconds. It consists of SBs resembling IIDs (Fig 4B and Fig 5), and each lasts hundreds of milliseconds. The intervals between IDs are about two minutes. The mechanism of IDs is given schematically in Fig 1B. The concentration [*K*]_*o*_ increases towards the level [*K*]_*bath*_. At some critical level of [*K*]_*o*_, the network begins to generate SBs. [*K*]_*o*_, the extracellular potassium concentration, increases during each SB and partially relaxes between SBs. The extracellular potassium accumulates (Fig 4A and 4C, blue line). The SB frequency rapidly grows with increasing [*K*]_*o*_, forming a cluster (Fig 4B and 4C). The intensified firing leads to an accumulation of sodium inside neurons (Fig 4A and 4C, red line). The Na-K-pump function strongly depends on [*Na*]_*i*_; therefore, the pump activity rapidly increases (Fig 4A and 4C, orange line). At some point, the pump dominates the outflux of potassium; thus, [*K*]_*o*_ begins to decrease. The decline of [*K*]_*o*_ decreases the frequency of SBs. The sodium concentration reaches its maximum and begins to restore. While [*Na*]_*i*_ is high, the Na-K-pump is highly active, which maintains the reduction of potassium concentration. At some level of [*K*]_*o*_, the SB generation terminates. During the next cycle, towards the next ID, [*K*]_*o*_ begins to increase (Fig 4A) due to the diffusion with the bath solution, which dominates at this stage.

The regime with IDs is obtained with relatively slow [*K*]_*o*_ relaxation (*τ*_*K*_ = 100*s*, in accordance with the basic parameter set) compared with the characteristic interburst interval. This slow relaxation leads to potassium accumulation at some point of the train of SBs. At some critical level of [*K*]_*o*_, depolarization due to the shift of *V*_*K*_(*t*) (term 1 in Eq 6) overcomes the decrease of the excitation due to the decrease of the synaptic resource *x*^*D*^(*t*). It begins an ID via the positive feedback provided by the depolarizing effect of the potassium accumulation.

The results of this subsection lead to the conclusion that Epileptor-2 is able to describe both regimes with interictal and ictal discharges. The mechanisms of the discharge generation correspond to those shown in the schematic in Fig 1B. Simulations illustrate crucial roles of each of the processes included in the schematic (S1 Text, section 1). Modifications of Epileptor-2, given in S1 Text, sections 2–4, extend the model to a case of epileptiform activity in a disinhibited network, a case of prominent depolarization block or consideration of more realistic, adaptive neuron-observer.

### Comparison of the model with the experiments

**Fig 6:**
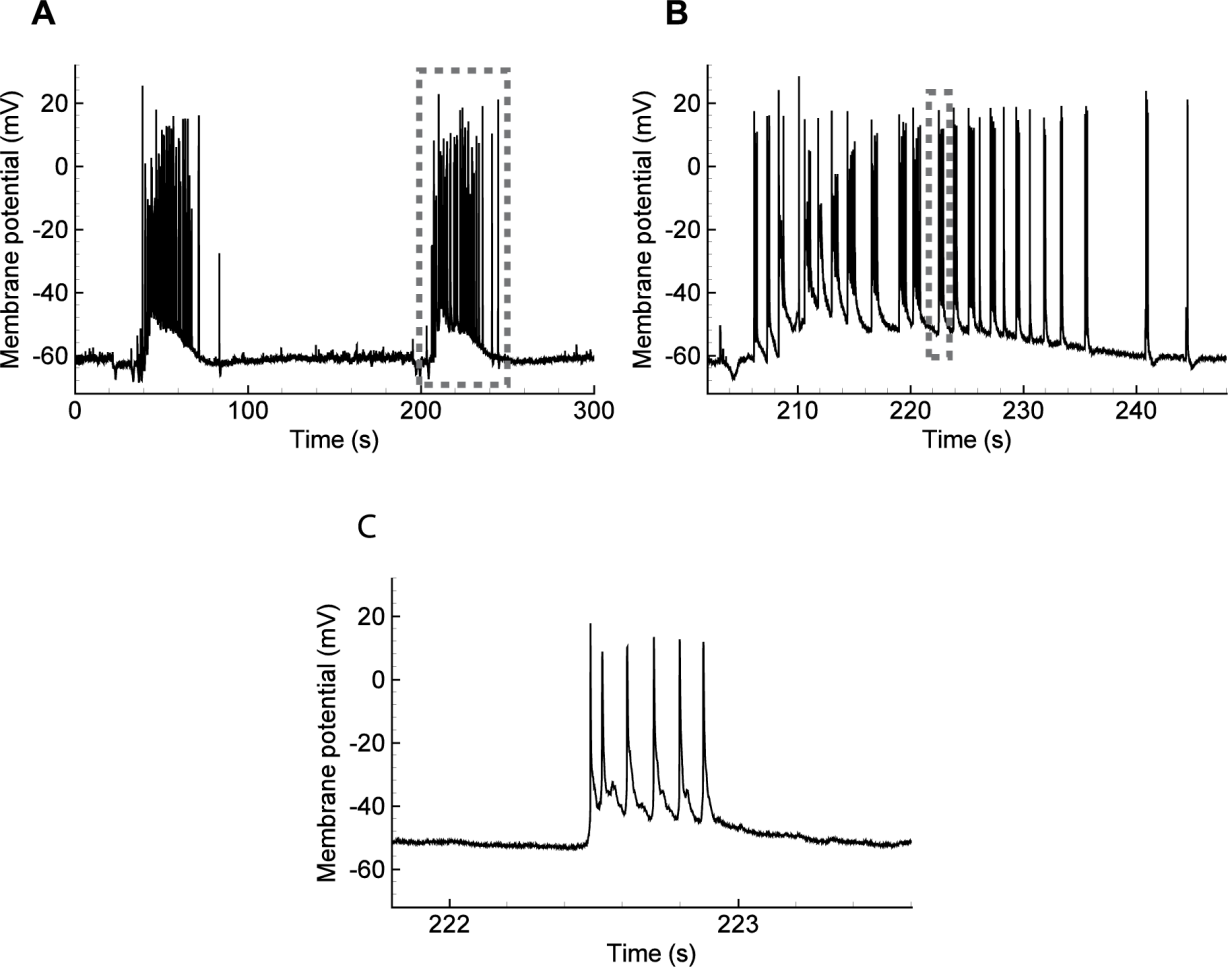
Ictal discharges recorded in a pyramidal neuron from a rat entorhinal cortex using an *in vitro* 4-AP model of epileptic activity. (**A**) The full record. (**B**, **C**) Zoomed fragments. Note each ID is a burst of SBs of spikes.

The IDs simulated using the proposed model are similar to the those registered in slices of a rat entorhinal cortex (Fig 6). The ictal events occurred with a period of about three minutes (compare Fig 6A with Fig 5A). Each ID consisted of SBs (Fig 6B with Fig 5B). Each SB consisted of several spikes (Fig 6C with Fig 5C). The shift of the baseline of the potential during an ID was observed during the experiment (Fig 6A) and in the average potential *V*(*t*) in the model (Fig 4A); it is not present in *U*(*t*) due to the approximate nature of the single neuron model used.

**Fig 7:**
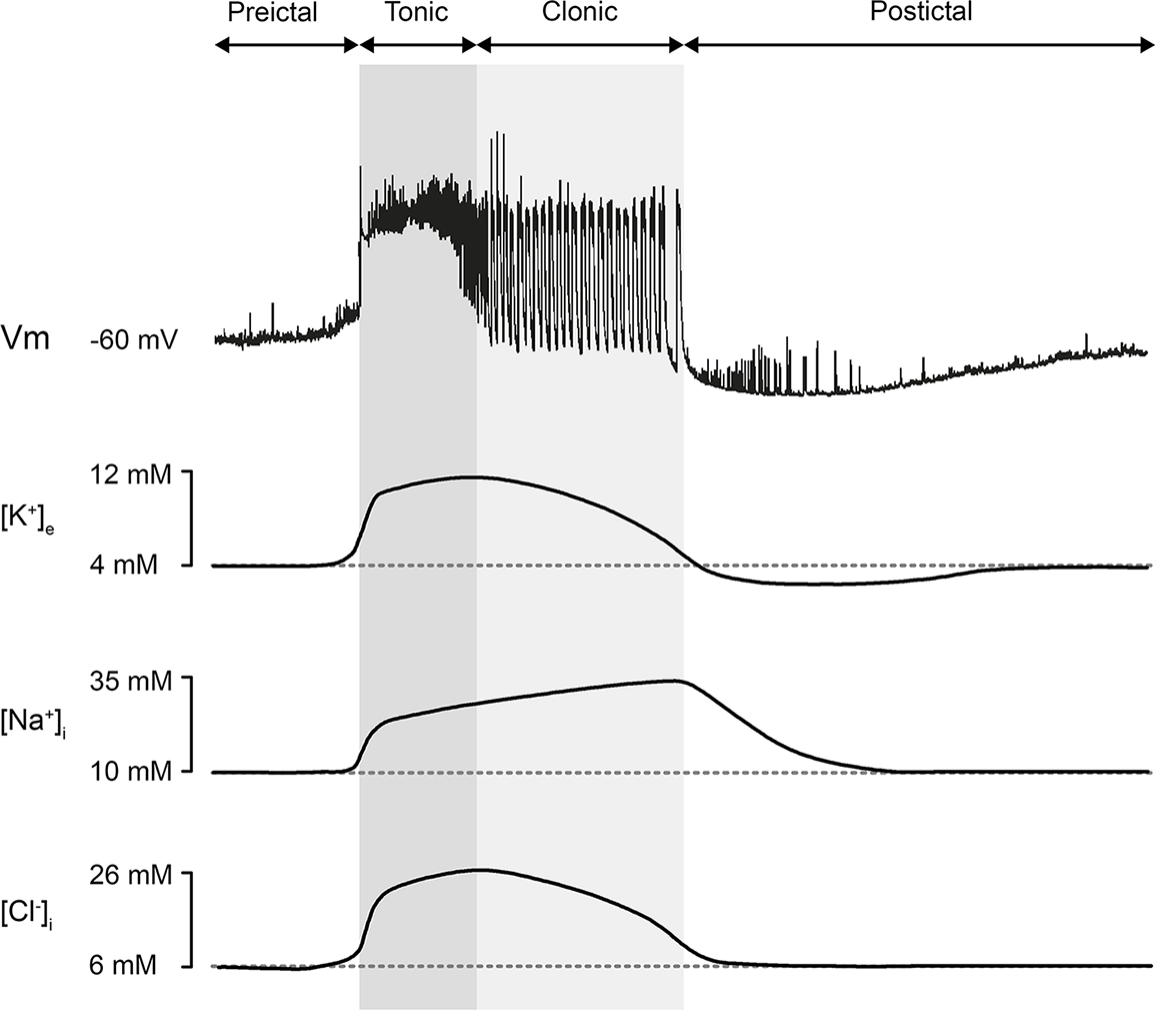
Ion concentration changes during seizures. Modified with permission from [15].

**Ionic dynamics.** The transmembrane concentration gradient of the six major ions (K^+^, Na^+^, Cl^-^, Ca^2+^, H^+^, and HCO_3_^-^) is altered during an epileptic seizure [15]. The most well-studied are the levels of extracellular K^+^ and Ca^2+^ and intracellular Cl^-^, Na^+^, and H^+^ concentrations [15]. Changes in the concentration of extracellular K^+^ and intracellular Cl^-^ and Na^+^ play the most critical roles in seizure induction and maintenance [15]. The central assumptions of the Epileptor-2 assert that only extracellular K^+^ and intracellular Na^+^ concentrations govern the activity, thus excluding intracellular Cl^-^ from consideration. This assumption implies a proportionality between the changes of [*K*]_*o*_ and [*Cl*]_*in*_. Fig 7 was reproduced from the study by Raimondo [15] and supports this assumption. The profiles of [*K*]_*o*_ and [*Cl*]_*in*_ during ID are similar. Presumably, this proportionality is explained by the activity of K-Cl-cotransporters, which evoke a potassium outflux in response to chloride accumulation within the neurons.

The extracellular potassium concentration increases during a single SB up to 1–2 mM according to data from [33] (see their Fig 7 or Fig 12 in [5]) and [8,14]. This potassium flux originates from both pyramidal cells and interneurons [8]. The potassium voltage-gated channels, glutamatergic receptors, and K-Cl cotransporters contribute to the flux. The increase of [*K*]_*o*_ during a single SB results in moderate depolarization on about 1 mV according to Eq 7. In the Epileptor-2, the value of parameter *δ*[*K*]_*o*_ provides the same level of potassium increase after each SB; the parameter *τ*_*K*_ defines the speed of potassium level relaxation, and the parameter *g*_*K*,*leak*_ controls the level of polarization dependent on [*K*]_*o*_.

According to the experimental observations [5,8,14,15], the extracellular potassium concentration has a peak in the middle of an ID, while the intracellular sodium concentration still gradually increases until the end of an ID [15] (Fig 7). The model reproduces the ionic dynamics (Fig 4A), employing the nonlinear activation of the Na-K-pump. The pump is activated by a high sodium concentration (Fig 4C) according to the approximation (Eq 8). The influx of potassium through the pump overcomes the outflux, and [*K*]_*o*_ begins to decrease. Note that the peak of [*K*]_*o*_ precedes the decrease of [*Na*]_*i*_ in the simulation, the mentioned experiment (Fig 7), and the previous modeling [9]. The decrease of [*K*]_*o*_ results in the termination of the ID, as shown by the experiments. The model explains this ID termination by the decrease of the potassium-dependent current (term 1 in the r.h.s. of Eq 6). This suggests a crucial role of the Na-K pump in ID termination.

Indirect support of the hypothesis of the crucial role of the ATP-dependent Na-K-pump in ID termination is provided by the evidence of a drop in the oxygen concentration during an ID, which reflects the activity of energy metabolism [1].

**Fig 8:**
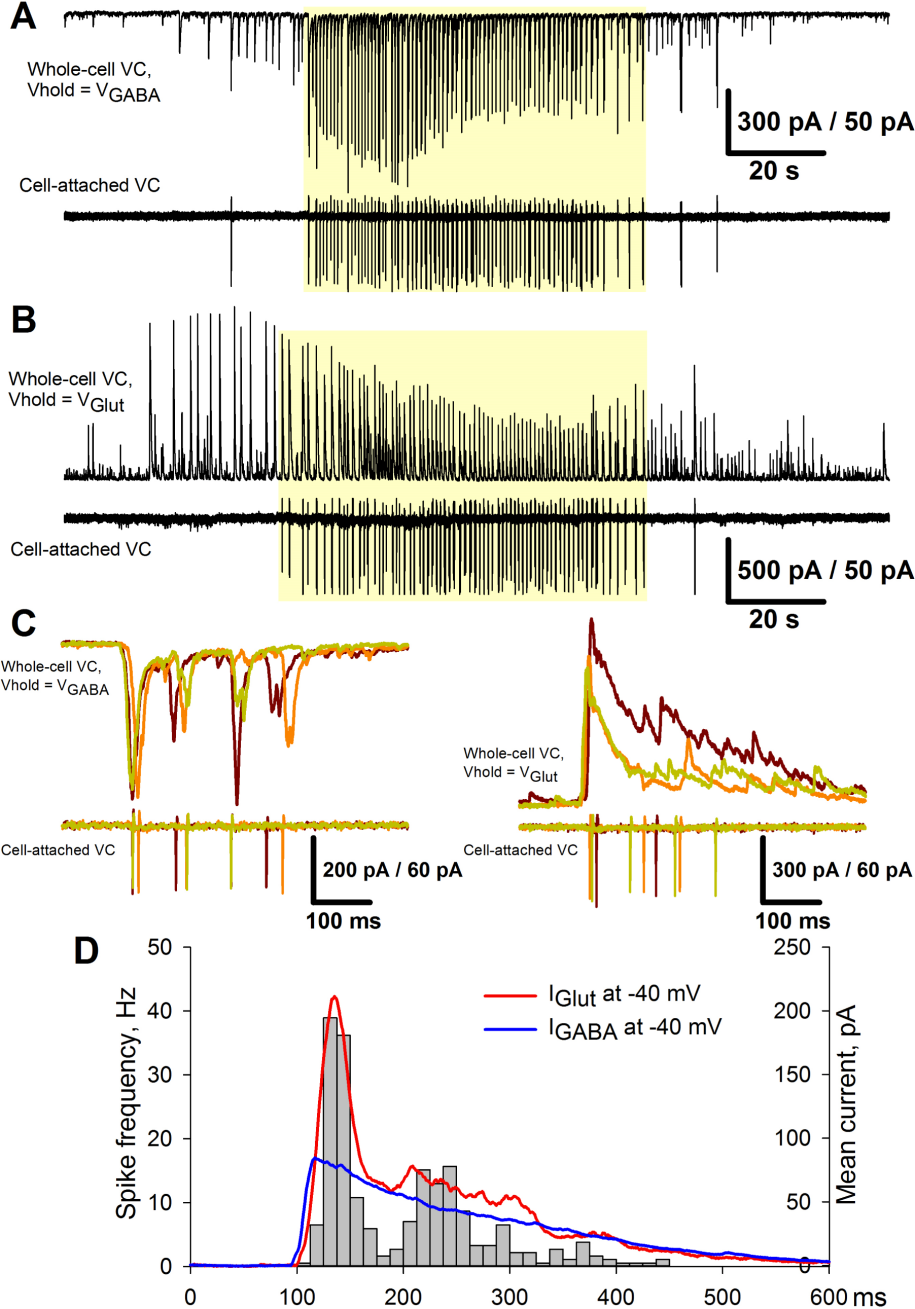
Proportionality between the excitatory and inhibitory currents and the firing rate in the experiment. (**A**) Upper trace: A representative recording of glutamatergic synaptic activity in the voltage-clamp mode at the reversal potential of GABA-mediated currents (*V*_*hold*_ = *V*_*GABA*_ = −51*mV*). Lower trace: A corresponding firing activity of the neighboring neuron recorded in the cell-attached voltage-clamp mode. (**B**) The recordings from the same pair of neurons as in A. The upper trace was recorded at the reversal potential of glutamate-mediated currents (*V*_*hold*_ = *V*_*Glut*_ = 0) and represents the GABAergic synaptic activity. The yellow shadow indicates the IDs. (**C**) Several superimposed IID-like events from A (left) and B (right) that correspond to SBs and constitute IDs. (**D**) Pooled data from four IDs, each consisting of 50–80 SBs as in C. A histogram represents the firing rate of the neuron recorded in the cell-attached VC mode. Solid lines are the plots of GABA- and glutamate-mediated currents scaled to represent the synaptic input at the membrane potential of -40 mV.

**Synaptic currents.** The second term in Eq 6 implies a proportionality between the excitatory and inhibitory currents and the firing rate. The experiments support this approximation. Assuming that two neighboring pyramidal neurons receive roughly identical synaptic input during epileptiform events [7], the pairing recordings were made from the neighboring neurons during IDs and IIDs. The excitatory and inhibitory currents from one neuron in a voltage-clamp mode and detected spikes in another neuron in a cell-attached mode were recorded (Fig 8A and 8B). The holding voltages in the voltage clamp corresponded to the reversal potentials of GABAergic and glutamatergic currents, -50 mV and 0 mV, respectively. The current profiles for several SBs were superimposed (Fig 8C). The mean currents were rescaled to correspond to the voltage level -40 mV, which is close to the spike threshold. These mean inhibitory and inverted excitatory currents are shown in Fig 8D. The instantaneous firing rate was calculated from cell-attached recordings as an average number of spikes per interval divided by the length of the interval (12.5 ms). It was found that (i) the shapes of the currents and the instantaneous firing rate are roughly similar; (ii) at spike threshold potential, the excitatory current is bigger than the inhibitory one; (iii) at the initial stage, the peak of the excitatory current is twice larger than that of the inhibitory current; and (iv) the excitatory current decreases faster than the inhibitory one. These findings support the assumptions of the proportionality of the currents to the firing rate, the effect of short-term synaptic depression, and the ratio of excitatory versus inhibitory currents before the action of depression. Therefore, these findings (observations) validate term 2 of Eq 6.

### Mathematical analysis of SBs

Based on the simulations of the system of Eqs 1–8 (Fig 4), the variables *x*^*D*^(*t*) and *V*(*t*) oscillate faster than the dynamics of [*K*]_*O*_ and [*Na*]_*i*_, producing the SBs. Thus, the subsystem of Eqs 3–5 was studied under the conditions of fixed ionic concentrations, i.e., with $V_{K}(t)=V_{K}^{0}$. The system of Eqs 3–5 is planar and stochastic with non-smooth r.h.s., as follows:

$$C\frac{dV}{dt}=-g_{L}\;V+G_{syn}\;\nu (t)\;(x^{D}(t)-0.5)+\sigma \;\xi (t)$$ (3’)

$$\frac{dx^{D}}{dt}=\frac{1-x^{D}}{\tau_{D}}-\delta x_{}^{D}\;x^{D}\;\nu (t)$$ (4’)

$$\nu (V(t))=\nu_{\max}\;{\left[\frac{2}{1+\exp\left[-2\left(V(t)-V_{th}\right)/k_{\nu }\right]}-1\right]}_{+}$$ (5’)

**Fig 9:**
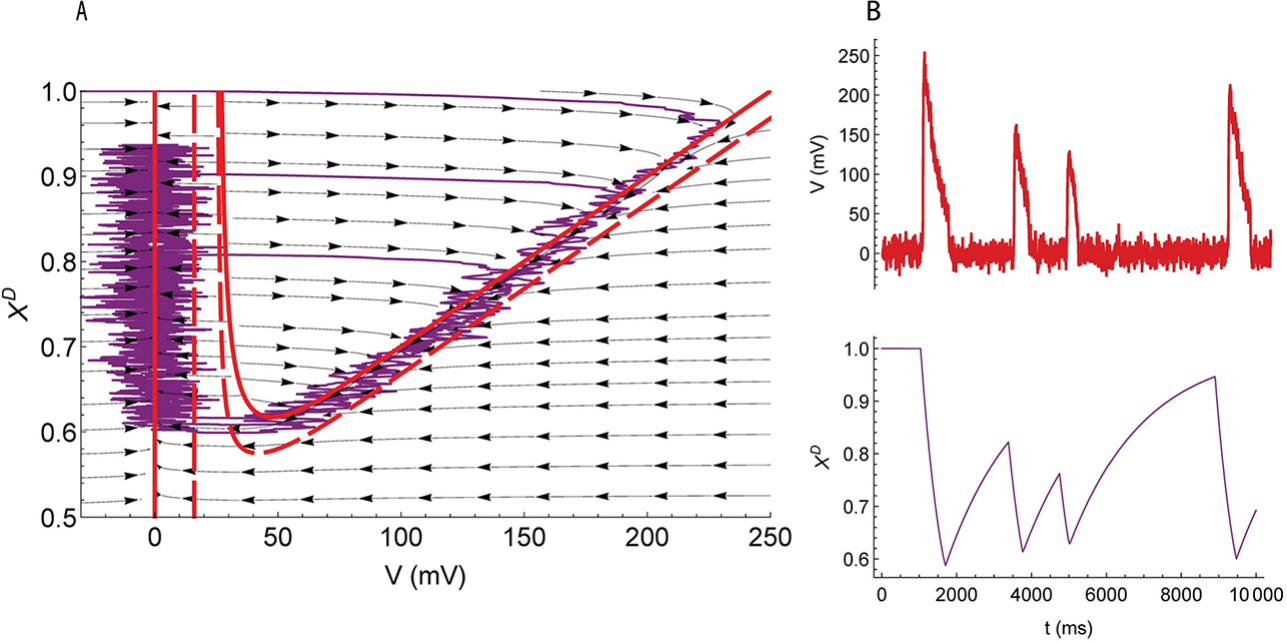
Fast subsystem producing SBs. (**A**) The phase space of the system of Eqs 3’–5’. Nullclines (solid red lines) and vector-fields (arrows) are calculated for ${\left[K\right]}_{O}^{}={\left[K\right]}_{O}^{0}$; dashed nullclines are for ${\left[K\right]}_{O}^{}={\left[K\right]}_{O}^{0}+10\;mM$. Purple curve is a trajectory. (**B**) the traces of *V*(*t*) and *x*^*D*^(*t*). The parameter values correspond to the basic set of parameters, except the noise amplitude *σ*/*g*_*L*_ = 40 *mV*.

Nullclines of the correspondent deterministic system (*σ* = 0) are shown in the phase space (Fig 9A). The deterministic system has a unique stable equilibrium point with *V* = 0 and *x*^*D*^ = 1. There are no other equilibrium points or limit circles. Thus, all possible trajectories converge to this stable steady state. Consider a trajectory with an initial position inside or to the left of the U-shaped nullcline. The trajectory first approaches the U-shaped nullcline, then reaches the bottom point, separates towards the straight line nullcline, and proceeds towards the steady state. Because a voltage trace that corresponds to this trajectory would be shaped as an impulse, the zones inside and to the left of the U-shaped nullcline could be referred to as zones of excitation.

Next, the effect of random disturbances on the system (3’-5’) that has the only attractor of the deterministic system was examined. A small amplitude noise that leads to small voltage fluctuations near 0 cannot excite the system. Indeed, small deviations from the equilibrium result in the monotonic convergence to the equilibrium; however, deviations larger than the threshold result in a large excursion before returning to the resting state. This implies a stochastic excitability. As the noise intensity increases, the time between two successive activations decreases. A random trajectory beginning from the stable equilibrium and declined by the noise to the zone of excitation is plotted in the phase plane (Fig 9A) along with corresponding time series (Fig 9B). When the noise intensity is higher than some threshold value, random trajectories can move far from the stable equilibrium, and large-amplitude oscillations occur along with small-amplitude oscillations. The intermittency of the oscillations forms the stochastic bursting.

Finally, the effect of the extracellular potassium concentration on the frequency and magnitude of SBs was examined. The level of [*K*]_*O*_ determines an additive current in Eq 3 according to Eqs 6 and 7. An increase in [*K*]_*O*_ leads to an inward current. The inward current shifts the U-shaped nullcline left and down and shifts the straight line nullcline to the right (dashed lines in Fig 9A). Consequently, the probability of the excursion in the phase-space to cross the nullcline from left to right is higher. The amplitude of oscillations decreases, and the frequency increases.

The noise brings a new quality to the system, which cannot be observed in the deterministic case. This phenomenon, known as coherence resonance, is a generic feature of noise-driven excitable systems and is mostly independent of the particular details of the model [16,34,35]. For epileptic discharges, the mechanism of stochastic large-amplitude oscillations underlies the SBs observed as interictal events and as primary components of IDs.

### Mathematical analysis of IDs as slow oscillations

To analyze an appearance of IDs, the full model was reduced to a slow subsystem. In the simulations (Fig 4), the dynamics of [*K*]_*o*_ and [*Na*]_*i*_ consisted of slow and low-amplitude fluctuations. The former are much slower than the dynamics of *x*^*D*^, *V*, and *ν*. Also, it was noted that Eqs 1 and 2 have slower time scales (*τ*_*K*_ and *τ*_*Na*_) than Eqs 3 and 4 with the time scales *τ*_*m*_ and *τ*_*D*_. These observations allowed for reducing the full model to a slow subsystem that is based on Eqs 1 and 2. The fast subsystem based on Eqs 3 and 4 and discussed in the previous subsection is controlled by the only slow variable [*K*]_*o*_. On the other hand, Eqs 1 and 2 are affected by the fast subsystem only through *ν*(*t*). In the simulations, the slow high-amplitude oscillations of [*K*]_*o*_ and [*Na*]_*i*_ did not follow the fast fluctuations of *ν* but were controlled by a slow component of *ν*. Therefore, to extract a slow subsystem, *ν*(*t*) was averaged, and the dependence of this average variable $\bar{\nu }$ on [*K*]_*o*_ was evaluated. This was done with the integration of Eqs 3–7 for the slow ramp of [*K*]_*bath*_ (from 3 to 22 mM during 200s) with the other parameters taken from the basic parameter set. The obtained dependence was approximated as follows:

$$\bar{\nu }\left({\left[K\right]}_{o}\right)=\left\{ \begin{array}{l} 0,\;if\;{\left[K\right]}_{o}<4.5;\;\;otherwise\\ -63.9093+20.0921{\left[K\right]}_{o}-1.53505\;{\left({\left[K\right]}_{o}\right)}^{2}\\ +0.0533615\;{\left({\left[K\right]}_{o}\right)}^{3}-0.000690027\;{\left({\left[K\right]}_{o}\right)}^{4} \end{array}\right.$$ (5”)

which is valid for [*K*]_*o*_ < 20 *mM*.

After the substitution of *ν* by $\bar{\nu }$ in Eqs 1, 2 and 8, the slow subsystem is obtained:

$$\frac{d{\left[K\right]}_{o}}{dt}=\frac{{\left[K\right]}_{bath}-{\left[K\right]}_{o}}{\tau_{K}}-2\gamma \;I_{pump}+\delta {\left[K\right]}_{o}\;\bar{\nu }({\left[K\right]}_{o})$$ (1’)

$$\frac{d{\left[Na\right]}_{i}}{dt}=\frac{{\left[Na\right]}_{i}^{0}-{\left[Na\right]}_{i}}{\tau_{Na}}-3\;I_{pump}+\delta {\left[Na\right]}_{i}\;\bar{\nu }({\left[K\right]}_{o})$$ (2’)

$$I_{pump}=\frac{\rho }{\left(1+\exp(3.5-{\left[K\right]}_{o}))(1+\exp((25-{\left[Na\right]}_{i})/3)\right)}$$ (8’)

where [*K*]_*bath*_ = 8.5 *mM*, ${\left[K\right]}_{o}^{0}=3\;mM$, ${\left[Na\right]}_{i}^{0}=10\;mM$ *τ*_*K*_ = 100 *s*, *τ*_*Na*_ = 20 *s*, *δ*[*K*]_*o*_ = 0.02 *mM*, *δ*[*Na*]_*i*_ = 0.03 *mM*, *γ* = 10, *ρ* = 0.2 *mM*/*s*, as in the basic parameter set.

**Fig 10:**
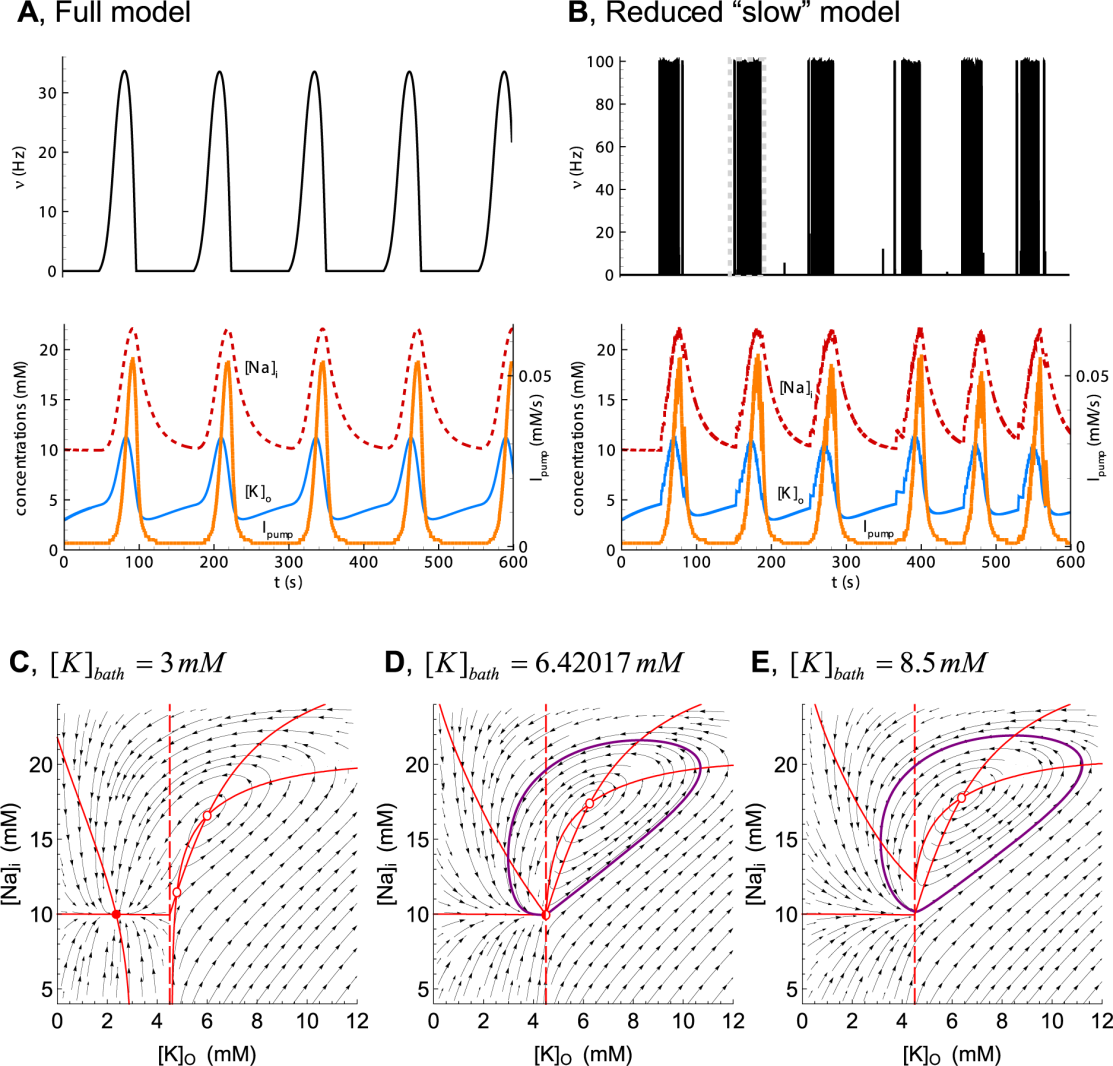
Slow subsystem producing IDs. (**A**) IDs in the full Epileptor-2 model. (**B**) Oscillations in the reduced model based on Eqs 1’, 2’, 5” and 8’. The plots repeat those in Fig 4. (**C**, **D**, and **E**) Phase portraits of the reduced model obtained for [*K*]_*bath*_ = 3, 6.42017, and 8.5 *mM*, correspondingly. Note the stable node in C and the limit cycle around the unsteady focus in D.

The reduced model based on Eqs 1’, 2’, 5” and 8’ behaves similarly to the full Epileptor-2, as shown in Fig 10.

The slow dynamical system was then analyzed based on Eqs 1’, 2’, 5” and 8’. Under “normal” conditions of a low potassium concentration in the bath solution, [*K*]_*bath*_ = 3 *mM*, the system converges to a fixed point, which is a stable node. The other two equilibrium points are an unstable focus and a saddle. At some critical value of ${\left[K\right]}_{bath}^{crit.}=6.42017\;mM$, the stable node couples with the saddle at the boundary that is given by a kink of the function $\bar{\nu }\left({\left[K\right]}_{o}\right)$, Eq 5”. This bifurcation is a codimension-two bifurcation in a piecewise-smooth system [36]. To the best of the authors’ knowledge, a full classification of the codimension-two bifurcations of non-smooth systems is still challenging, even in the case of planar systems. Nevertheless, the bifurcation of this system is similar to SNIC (saddle-node on invariant cycle) bifurcation, which can be observed in smooth systems. In this bifurcation, a limit cycle originates around the unsteady focus when the node and saddle disappear. The limit cycle has a zero initial frequency and a finite amplitude. This bifurcation corresponds to the transition to ID generation in the Epileptor-2 model. In the pathological condition of ${\left[K\right]}_{bath}^{}>{\left[K\right]}_{bath}^{crit.}$, such as in the case of [*K*]_*bath*_ = 8.5 *mM*, as shown in Fig 4, a stable oscillation of [*K*]_*o*_ is observed, which controls the generation of SBs during IDs. Overall, the analysis explains the dynamics of the regime with ID generation due to oscillations of potassium and sodium concentrations, with each ID composed of clustered SBs.

## Discussion

### Model assumptions

In the present work, a mathematical model of IDs and IIDs is proposed based on a consideration of only a few dominating processes that underlie the generation of the discharges. These processes have been described in the form of a simple mathematical model consisting of four ordinary differential equations written in terms of physically meaningful variables, referred to as Epileptor-2, as an alternative to the well-known but more abstract model Epileptor [1]. Along with a modeled neuron-observer, simulations of Epileptor-2 provide voltage, firing rate, and ionic concentration traces that are close to experimental recordings in many aspects. The model has been derived under the strong assumptions described in the Results section. The most important assumptions are: (i) ion concentrations that affect the discharges are the extracellular potassium and the intracellular sodium concentrations (Fig 1A); (ii) both excitatory and inhibitory currents are mainly controlled by the firing rate of an integrated neuronal population; (iii) only potassium concentration affects the intrinsic neuronal excitability; (iv) the excitatory and inhibitory currents are controlled by the same presynaptic firing rate; and (v) the excitatory current undergoes short-term depression.

The first assumption has been partially validated by experimental observations from the literature [15]. It is also consistent with that from modeling studies [10,11,32]. For instance, it was concluded in the detailed modeling study that it is the coupled dynamics of sodium, potassium, and chloride ions play a critical role in the development and termination of seizures [32]. The main ground for this assumption was relatively high concentrations of the complementary intracellular potassium, and extracellular sodium and chloride ions that are less affected by the ion dynamics and less affect the reversal potentials. Extracellular calcium concentration is shown to be progressively reduced during seizure [36], which decreases the reliability of synaptic transmission and can affect long-range synchronization properties of cell firing during ID [37]. Nevertheless, the mechanism of ID termination explored in this study depends on the specific cellular properties, such as activation of the Na-K pump. This pump is driven by ATPase and represents an intrinsic factor that is not directly affected by the extracellular calcium [32]. The intracellular calcium is connected to the neuronal excitation and short-term synaptic depression. Thus it implicitly affects the input-output function, Eq 5, and the parameters of Eq 4 for the synaptic resource. Based on these arguments the calcium ions are excluded from explicit consideration. In addition, our study associates the intracellular chloride concentration changes with the extracellular potassium, thus keeping only the extracellular potassium and the intracellular sodium concentrations.

The second assumption seems to be too strong if taking the sometimes crucial role of GABAergic interneurons in interictal discharges into account [6,7,22,31,38,39]; however, the types of the discharges studied more often include strong glutamatergic excitation [5,40] or are only based on glutamatergic interactions [23]. Meanwhile, even GABAergic channel-based IIDs are in agreement with Epileptor-2 if they are considered contributors to excitatory but not inhibitory currents, which are described by the second term in the r.h.s. of Eq 6. Indeed, GABA-based excitation implies a depolarized GABA receptor reversal potential. In this case, quantitatively, these receptors contribute to the positive term. The synapses that maintain a healthy level of chloride contribute to the negative term. Interneurons that are presynaptic to both subpopulations of synapses are active simultaneously. Thus, both synaptic currents roughly correlate with the firing rate of the excitatory population, as supposed by the fifth assumption. These arguments extend the applicability of the Epileptor-2 to the case of interneuron-driven discharges.

The third assumption postulates an essential role of the extracellular potassium concentration. It corresponds to the Fertziger and Ranck hypothesis [41]; however, the intracellular chloride concentration is also a potent regulator of neuronal excitability. These two variables are tightly connected, as the accumulation of intracellular chloride due to the activation of GABAergic receptors leads to an increase in KCC2-activity and a consecutive increase in extracellular potassium levels, which in turn directly affects the excitability and results in a less-efficient evacuation of chloride ions from the cell. The latter might at some point result in a failure of inhibition, causing more dramatic effects on neuronal excitability. In some experimental models of epilepsy, the activity of GABAergic interneurons is the primary cause of extracellular potassium build up and the progressive increase of neuronal excitability [5,6]; however, in this model, no particular cell types were introduced, and the firing rate was assumed to be the same for all cell types. A feasible approximation in this case is an assumption of the proportionality between the elevation of extracellular potassium and intracellular chloride concentrations, which allows for accounting for only one type of ion. Thus, in this model, several steps that link firing activity to potassium accumulation were skipped, and a simplified approach where cell firing directly affects the potassium concentration was implemented. Moreover, the change in the level of potassium affects neuronal depolarization via the potassium reversal potential [42]. Therefore, for simplicity, only the potassium concentration was included in the model, and the proportionality between intracellular chloride and extracellular potassium concentrations was assumed. Based on this assumption, the effects of their temporary imbalance [43] was neglected. Consequently, this approach is entirely different from that by Proix et al. [44], who introduced a permittivity variable that captures effects evolving on slow timescales and including extracellular ionic concentrations and energy metabolism. The permittivity variable controls the remainder of the dynamical system of the Epileptor. The advantage of the new approach is the explicit form of the dependence on the potassium concentration.

The fourth assumption has been validated based on the experimental data. More precise matching would require explicit consideration of excitatory and inhibitory populations synaptic components to fit experimental estimates of either cumulative excitatory and inhibitory synaptic conductances [45,46] or AMPA, NMDA and GABA-mediated conductances, as done in [31] for interictal discharges. However, such detailed modeling in the case of ID generation would involve a complex description of the ionic dynamics. Thus such modeling is on the opposite extreme of detail in comparison to the proposed Epileptor-2 model. Another critical point to the fourth assumption is its validity in the case of the depolarization block, which occurs in interneurons during IDs [1,47]. Therefore, the approximation might overestimate the inhibitory currents as well as the potassium accumulation mediated by the accumulation of chloride and the activity of K-Cl-cotransporters during some period within an ID. These effects are expected to influence the duration of IDs. This view is supported by a modified Epileptor-2 model that takes into account the depolarization block, as described in S1 Text, section 3.

The fifth assumption is based on experimental evidences of a short-term depression of glutamatergic synapses [23,48,49]. An enhanced depression of GABAergic synaptic transmission was also found to be a crucial factor for reproducing synchronized synaptic activity in the previous model of interictal discharges. Experimental observations partly confirmed this assumption, as GABA-mediated responses to extracellular stimuli seemed to be occluded [31]. The synaptic depression of inhibitory synapses has not been included in the model for simplicity because its contribution could only reshape the discharges.

In summary, the assumptions of the Epileptor-2 model are in line with a wide range of experimental evidence obtained in different models of epilepsy.

### Stochastic oscillations as a mechanism of epileptic discharges

Based on the simulations, both ictal and interictal discharges consist of elementary events, SBs. To the best of the authors’ knowledge, the IDs have not been modeled before as bursts of spike bursts. The SBs have been mathematically analyzed. They were found to be large amplitude stochastic oscillations. The second-order system of differential equations is probably the simplest model of these kernel elements of pathological discharges. This statement is an important prediction, which opposes the inherently stochastic mechanism of the discharge generation to an oscillatory mechanism. The latter explains a stochastic sequence of the discharges due to noise in a deterministic periodic process. In contrast, the former implies that the discharges are impossible without noisy fluctuations. Similar behaviors have been studied for a neuronal model [16].

### Bifurcations leading to epileptic discharges

The original Epileptor model explains epileptic discharges in terms of bifurcations [1]. In this mode, the types of bifurcations that correspond to the onset and offset of IDs are saddle-node and homoclinic bifurcations, respectively [1] with the control parameter of “permittivity,” which is the slowest variable. The saddle-node bifurcation at the onset of IDs in the Epileptor was chosen based on experimentally observed features, such as fixed frequency and fixed amplitude of abruptly started oscillations, and a shift of baseline field potential. The homoclinic bifurcation at the offset of IDs was chosen for the shift of the baseline, fixed amplitude, and decaying frequency of oscillations.

In contrast to the permittivity parameter in the Epileptor, this model has [*K*]_*O*_ as the control variable of fast oscillations, SBs. The variable [*K*]_*O*_ shows slow oscillations with pulses (Fig 10A and 10B). Due to these oscillations, the frequency of SBs oscillates as well, forming IDs; however, no bifurcation occurs in the fast, stochastic system. Instead, the frequency of SBs changes along with the probability of spontaneous SB generation, depending on the level of [*K*]_*O*_, as explained with the mathematical analysis. The three characteristic features of the onset of ID are similar to the ones in the original Epileptor: (i) the amplitude of the fast oscillations is large (Fig 9); (ii) their frequency grows not abruptly but rapidly (Fig 4B) due to the rapid onset of the pulses of [*K*]_*O*_; and the baseline shift of voltage (Fig 4A) occurs due to the depolarizing effect of the high level of [*K*]_*O*_. The offset of ID corresponds to the decaying phase of a slow pulse of [*K*]_*O*_ with a rate of frequency decrease similar to that at the onset of ID. Thus, to a large extent, the Epileptor-2 reproduces the main features of IDs that were captured by the original Epileptor.

The analysis explains the scenario of an origination of a regime of ID generation during a change in an external factor, an increase of [*K*]_*bath*_, similar to previous studies conducted with a single neuron model [9,10]. Using a fast-slow analysis, it was found that the scenario corresponds to the SNIC-like bifurcation. This implies that: (i) IDs are seldom at the levels of [*K*]_*bath*_ just above the critical value and (ii) the amplitude of [*K*]_*O*_ oscillations is always finite, which indicates a similar shape of IDs at different [*K*]_*bath*_. Both predictions are more or less consistent with the experimental observations. The whole system of the Epileptor is of fold/homoclinic type [50].

The Epileptor-2 can not be classified in the same way. Instead, fast and slow dynamics are to be considered separately. In contrast to the Epileptor, the Epileptor-2 is a non-smooth, stochastic dynamical system. The non-smoothness is due to the kink in Eq 5”. This kink reflects the threshold nature of neuronal excitation. This is consistent with the observations of the rapid development of spontaneous spiking in a slice that undergoes a change in potassium concentration from low to high values in a bath. The presence of noise in the Epileptor-2 is also quite natural and realistic. The voltage fluctuation in normal and pathological states and an irregularity of IIDs indicate the intrinsic stochasticity of the network, which validates the predictions of the Epileptor-2 model.

Several mechanisms of seizure recruitment have been tested in computational models with varying levels of mathematical abstraction [32,52,53]. Typically, the most complex, spatially distributed, stochastic models are studied by means of their reduction to the system of ordinary differential equations. For example, the model proposed by Steyn-Ross et al [54] has been modified and studied by Kramer et al. [51,55]. Their main variable is compared to the experimentally registered local field potential. The oscillating regime for this variable is regarded as an ictal state. The transitions to and from the ictal state have been studied in a plane of two parameters of excitation. The obtained bifurcations help to understand the behavior of the full spatially distributed, stochastic system. In comparison with the Epileptor-2, the ionic dynamics is not considered in the Kramer’s model, however the excitation variables might be related to the ionic concentrations. In the regime of ID generation in Epileptor-2, the concentrations oscillate. Therefore, the transitions in the Kramer’s model are to be compared to the gradual change of SB generation in Epileptor-2, from the rare bursting between IDs to the fast bursting during each of the IDs. In Epileptor-2, there are no bifurcations. Instead, the probability of the SB generation gradually changes, with SBs being described by the fast subsystem and always representing stochastic large-amplitude oscillations. In this sense, our model is qualitatively different from the previous models [32,43,52,53]. Meanwhile, in the Epileptor-2, the slow dynamics of the concentrations undergoes bifurcations of the transitions to and from the oscillations governed by one of the external parameters of excitation, like the bath potassium concentration that controls the SNIC-like bifurcation.

### Conclusion

In this paper, a simple model of epileptic discharges is proposed, and the fundamental mechanisms described by this model are illustrated. The model is qualitatively and in a sense quantitatively compared to a wide range of experimental recordings. Similar to the known Epileptor model, this model can be applied to investigate the pathological states in a network of coupled oscillators [44]. It can also be used as a navigator in the parameter space for biophysically detailed modeling.

## Methods

### Ethics statement

All animal procedures followed the guidelines of the European Community Council Directive 86/609/EEC and were approved by the Animal Care and Use Committee of the Sechenov Institute of Evolutionary Physiology and Biochemistry of the Russian Academy of Sciences.

### Slice preparation and electrophysiological recordings

Experimental procedures were described previously in details in our recent paper [7]. Shortly, 3-week-old Wistar rats were decapitated and their brains removed rapidly. A vibrating microtome (Microm HM 650 V; Microm; Germany) was used to cut 300-μm thick horizontal slices that contained entorhinal cortex and hippocampus. Artificial cerebrospinal fluid (ACSF) was used through all steps, which had the following composition (in mM): 126 NaCl, 2.5 KCl, 1.25 NaH_2_PO_4_, 1 MgSO_4_, 2 CaCl_2_, 24 NaHCO_3_, and 10 dextrose. The ACSF was aerated with carbogen (95% O_2_/5% CO_2_). Recordings were made at 30˚C. Pyramidal neurons in deep layers of the entorhinal cortex were visualized using a Zeiss Axioscop 2 microscope (Zeiss; Oberkochen, Germany) equipped with a video camera (PointGrey Grasshopper3 GS3-U3-23S6M-C, FLIR Integrated Imaging Solutions Inc., USA) and differential interference contrast optics. Patch electrodes (3–5 MΩ) were pulled from borosilicate filamented glass capillaries (WPI; UK) on a P-1000 Micropipette Puller (Sutter Instrument; Novato, CA, USA). For current-clamp recordings, a potassium-gluconate-based filling solution was used with the following composition (in mM): 135 K-gluconate, 10 NaCl, 5 EGTA, 10 HEPES, 4 ATP-Mg, and 0.3 GTP (with pH adjusted to 7.25 with KOH). For voltage-clamp recordings, a solution based on cesium-methane-sulfonate (CsMeS) was used with the following composition (in mM): 127 CsMeS, 10 NaCl, 5 EGTA, 10 HEPES, 6 QX314, 4 ATP-Mg, and 0.3 GTP (with pH adjusted to 7.25 with CsOH). For cell-attached voltage-clamp recordings, a sodium-chloride-based filling solution was used. It had the following composition (in mM): 138.5 NaCl, 8.5 KCl, 10 HEPES, 5 EGTA (with pH adjusted to 7.25 with NaOH). Cell-attached voltage-clamp recording of neuron firing activity was performed as described [56].

Recordings were performed with two Model 2400 patch-clamp amplifiers (AM-Systems; Sequim, WA, USA), and an NI USB-6343A/D converter (National Instruments; Austin, TX, USA) using WinWCP5 software (SIPBS; Glasgow, UK). The data were filtered at 10 kHz and sampled at 20 kHz. After formation of the whole-cell configuration, access resistance was less than 15 MΩ and remained stable (< 30% increase) during the experiments in all cells included.

Epileptiform activity was induced with the pro-epileptic solution, containing the following (in mM): 120 NaCl, 8.5 KCl, 1.25 NaH_2_PO_4_, 0.25 MgSO_4_, 2 CaCl_2_, 24 NaHCO_3_, 10 dextrose, and 0.05 4-AP. The flow rate in the perfusion chamber was 5–6 ml/min. The liquid junction potentials were measured as described [57], and the holding potential was compensated offline for whole-cell voltage-clamp recordings by subtracting 7 mV.

### Modeling and analysis

The new model is based on the previous biophysically detailed considerations of ionic dynamics during the pathological states of brain activity [9,17,30,42]. The ionic dynamics were implemented in a rate-based model for recurrently connected excitatory and inhibitory neuronal populations, where the inhibitory population has been accounted for implicitly, and the firing rate was assumed to be proportional to that of the excitatory population. The firing rate has been described as a rectified sigmoid function of a membrane potential (Fig 2). The membrane potential has been described by Kirchoff’s current conservation law, which was written for a one-compartment neuron. The expressions for the excitatory and inhibitory synaptic currents, the input-output-function, the rate-based equations for the ionic dynamics, etc., have been justified in the Results section. The short-term synaptic depression is described according to the Tsodyks-Markram model [27]. A quadratic integrate-and-fire model was used as a model for a representative neuron [19].

The simulations were performed in the Delphi-7 environment. The mathematical analysis of the stochastic oscillations was performed using Wolfram Mathematica 10 (Champaign, IL, USA). The Euler-Maruyama explicit numerical scheme was applied for the integration of the stochastic ordinary differential equations. The typical value of a time step was 0.5 ms. The results were dependent on the numerical parameter in a similar extent as for different realizations of noise. The numerical realizations of the model are available from the websites: the code in Wolfram Mathematica is at https://yadi.sk/d/927UjbS-3QQhMW; the code and executive file in Delphi-Pascal are at https://drive.google.com/file/d/1AJhAFKLOjvgauBF_6SQzvol8zzmfMaDV/view?usp=sharing and https://drive.google.com/open?id=10ij-Nt780jROcMv9qniUm4rAJNr8WD2j, correspondingly.

## Supporting information

S1 Text Illustrations to the mechanisms of IDs and IIDs. Disinhibition as a model of epilepsy. Depolarization block. Alternative model of a neuron-observer.
(PDF)
