# Correction: Minimal model of interictal and ictal discharges “Epileptor-2”

**DOI:** 10.1371/journal.pcbi.1007359

**Published:** 2019-09-12

**Authors:** Anton V. Chizhov, Artyom V. Zefirov, Dmitry V. Amakhin, Elena Yu. Smirnova, Aleksey V. Zaitsev

In Fig 10 panels A and B are the wrong way around and the labels were therefore incorrect. The authors have provided a corrected version here.

**Fig 10:**
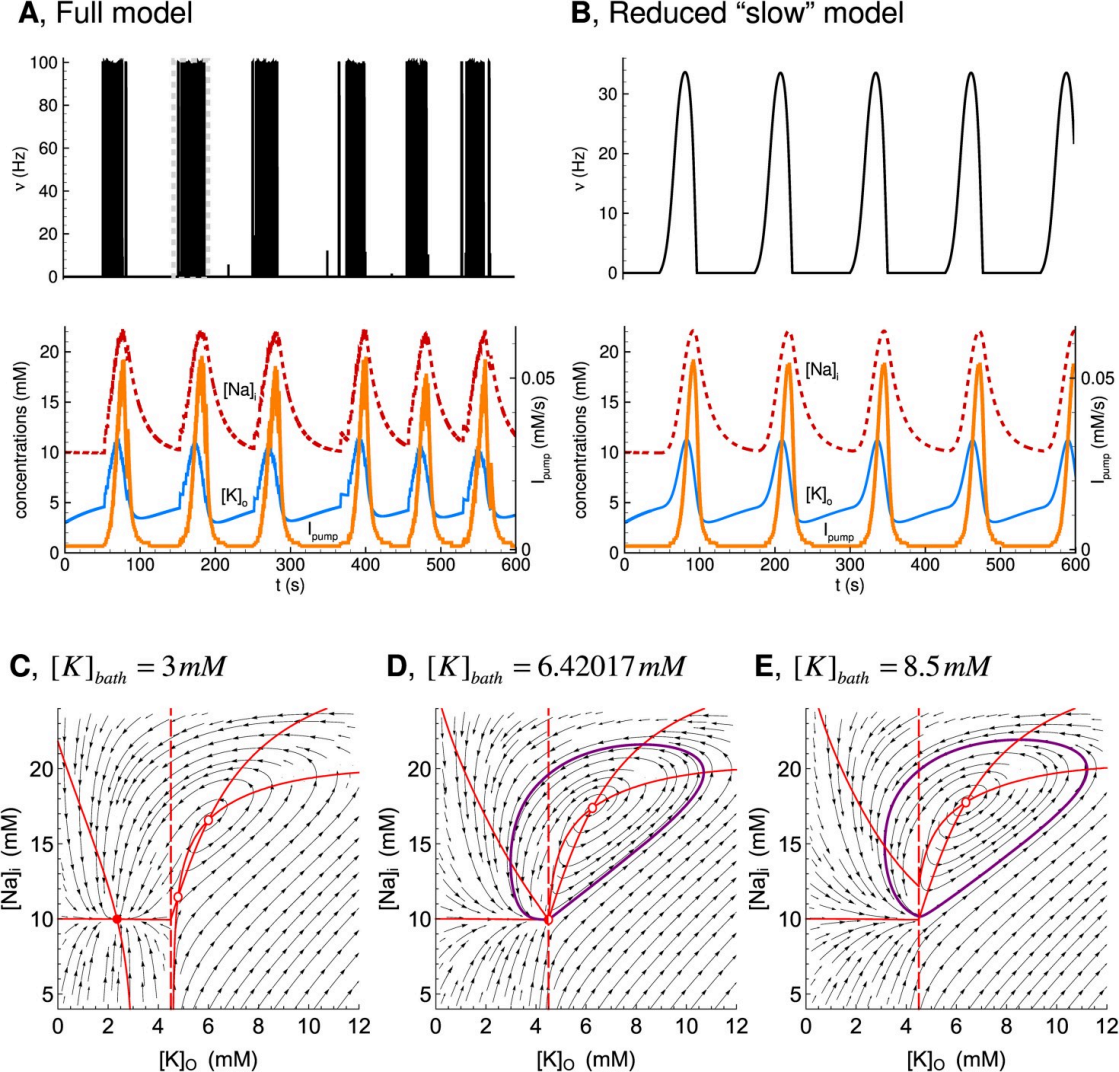
Slow subsystem producing IDs. (A) IDs in the full Epileptor-2 model. (B) Oscillations in the reduced model based on Eqs 1’, 2’, 5” and 8’. The plots repeat those in Fig 4. (C, D, and E) Phase portraits of the reduced model obtained for [K]bath = 3, 6.42017, and 8.5 mM, correspondingly. Note the stable node in C and the limit cycle around the unsteady focus in D.
