# Supplementary material for: Minimal model of interictal and ictal discharges “Epileptor-2”
Source: PLoS Comput Biol. 2018 May 31;14(5):e1006186. doi: 10.1371/journal.pcbi.1006186 (PMC6005638; doi:10.1371/journal.pcbi.1006186)
Supplement: S1 Text — Disinhibition as a model of epilepsy. Depolarization block. Alternative model of a neuron-observer. (PDF) [file pcbi.1006186.s001.pdf]

Supplementary material
to the paper by A.V. Chizhov, A.V. Zefirov, D.V. Amakhin, E.Yu. Smirnova and A.V. Zaitsev
(PLoS Computational Biology)

1. Illustrations to the mechanisms of IDs and IIDs. 2
2. Disinhibition as a model of epilepsy. 10
3. Depolarization block. 11
4. Alternative model of a neuron-observer. 12
References.. 13

1. *Illustrations to the mechanisms of IDs and IIDs.*

Below we illustrate the contribution of each of the elements of the mechanisms of simulated IDs and IIDs. The schematic of the mechanisms is given in Fig.1B in the main text and reproduced in Fig. A with the all the transitions (arrows) numbered.

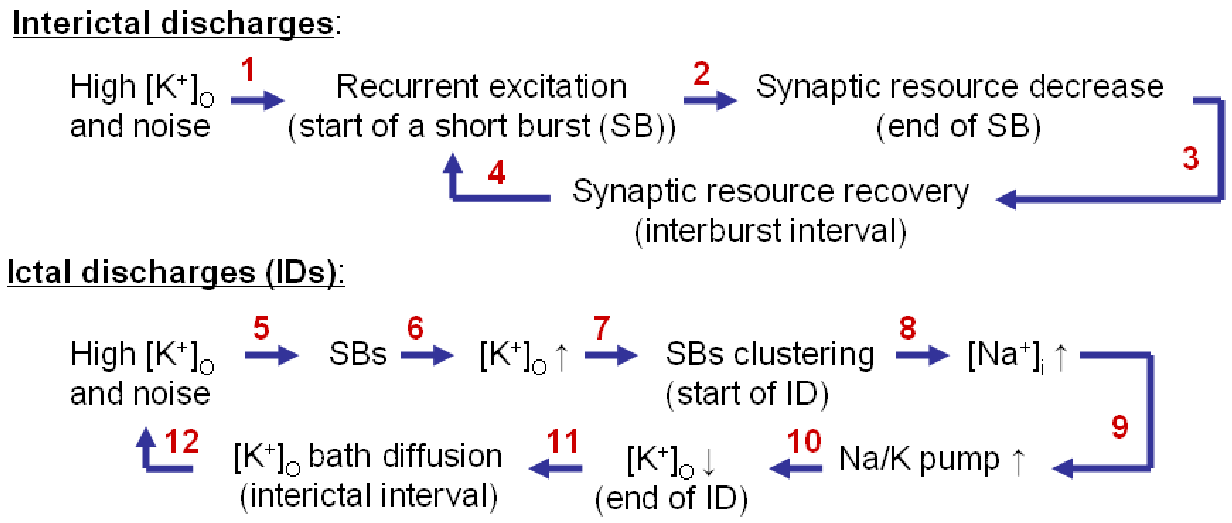

Fig. A. Mechanisms of interictal and ictal discharges (IIDs and IDs) (Fig. 1B in the main text). SBs are the short bursts or IID-like events.

According to the arrows in Fig. A, the main processes in the mechanisms of ID and IID generation are as follows:

1. High potassium concentration leads to recurrent excitation. A low potassium concentration results in a trivial, silent resting state of the system, which turns to IID generation regime when the bath concentration changes from 3 to 8.5 mM (Fig. B). At the interval between 50 and 100 ms a cluster of SBs is observed with significant modulation of $[K]_O$ and $[Na]_i$, similar to an ID, which then turns into irregular SBs generation with low modulation of the concentrations, i.e. to the regime with IID generation.

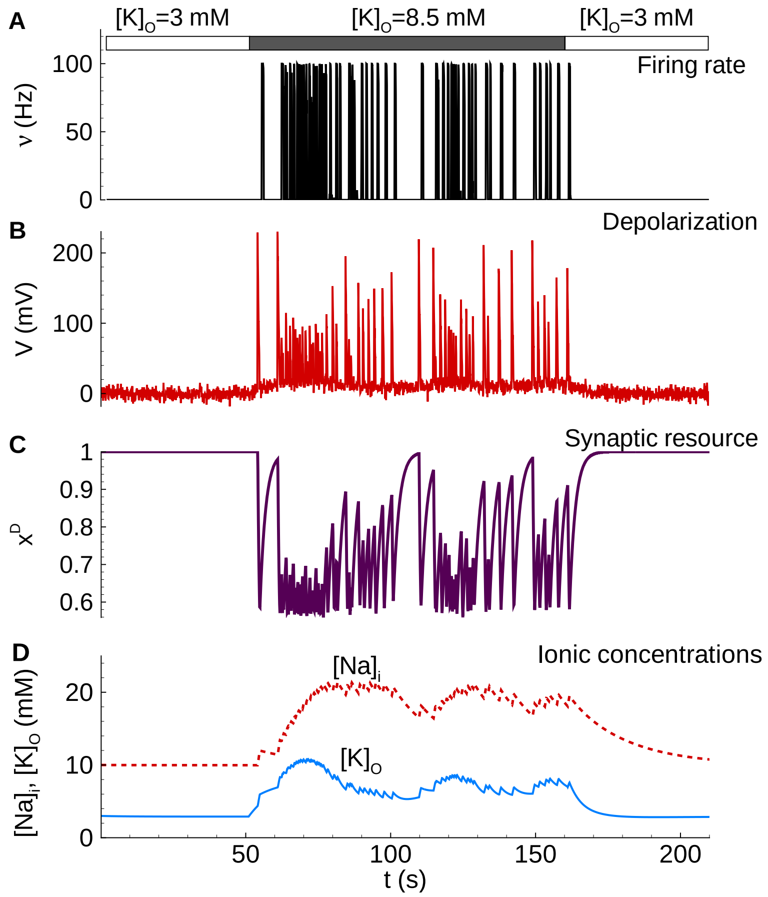

Fig. B. The transition from the resting state at $[K]_{bath} = 3\,mM$ to the IID generation ($\tau_K = 10\,s$) at $[K]_{bath} = 8.5\,mM$ at the time moment $t = 50\,s$ and back at $t = 160\,s$. The legend is in Fig. 3 of the main text.

2. SBs lead to the decrease of the synaptic resource, as seen in Fig. 3C in the main text. This is illustrated by the effect of the efficiency of the short-term synaptic depression δx^D. Reduction of the synaptic depression results in longer IID duration, as seen in Fig. C. Besides, the IDs tend to cluster.

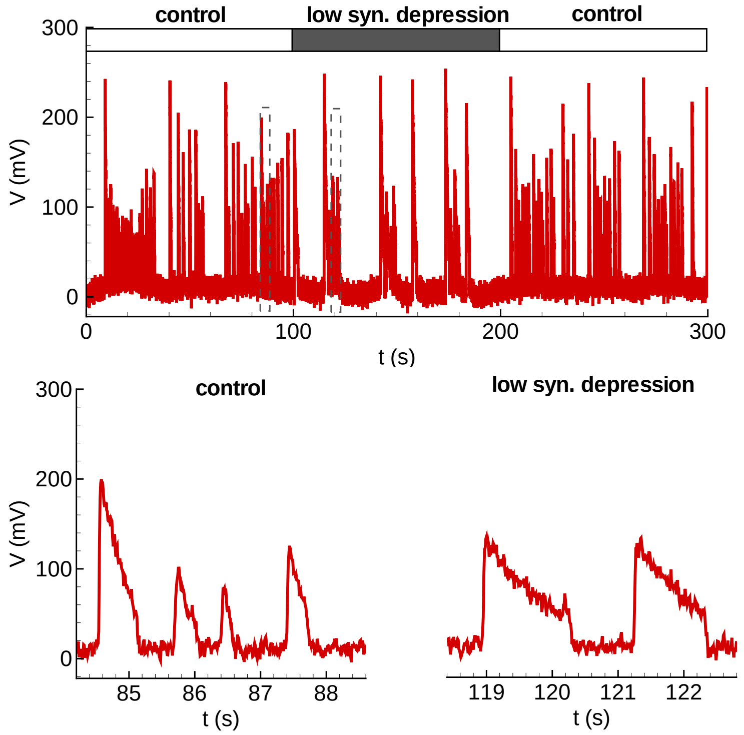

Fig. C. Reduction of synaptic depression results in longer IID duration. The parameter of synaptic depression efficiency δx^D was changed from its control value 0.01 to 0.005 at the time moment $t = 100\,s$, and returned at $t = 200\,s$.

3. The synaptic recovery leads to a new SB generation. As seen from Fig. D, the frequency of IIDs decreases with the increasing time of synaptic recovery, τ_D.
4. Naturally, the synaptic recovery after SB gradually increases the synaptic resource. The resource determines a synaptic conductance in response to excitation by the noise, thus increasing the probability of a new SB generation.

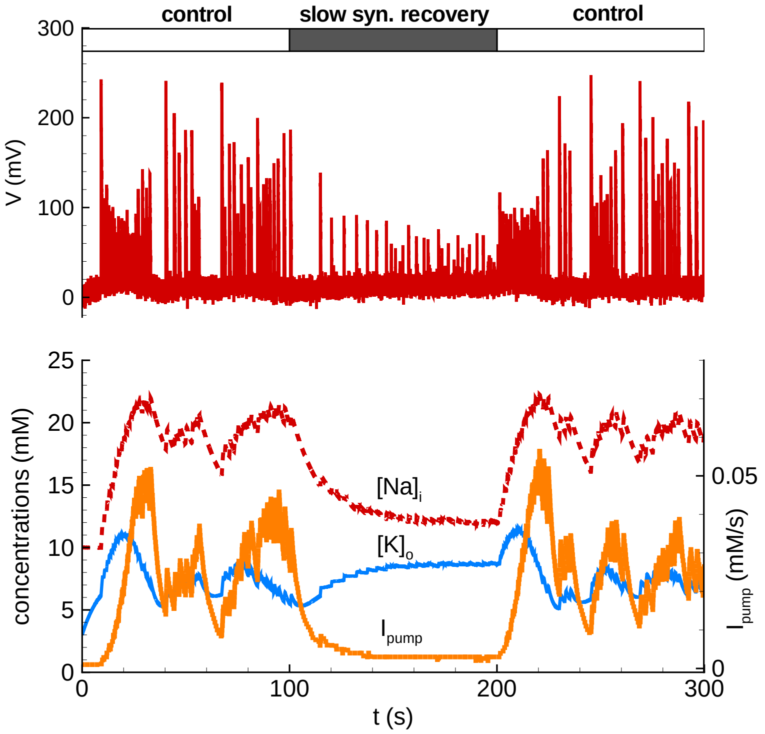

Fig. D. An increase of synaptic recovery time results in the increased frequency of IIDs. The parameter τ_D was changed from 2 to 20s, and back.

5. High potassium concentration leads to the recurrent excitation and consequently to SB generation. For example, an increase of the potassium concentration in a bath results in ID generation (Fig. E).

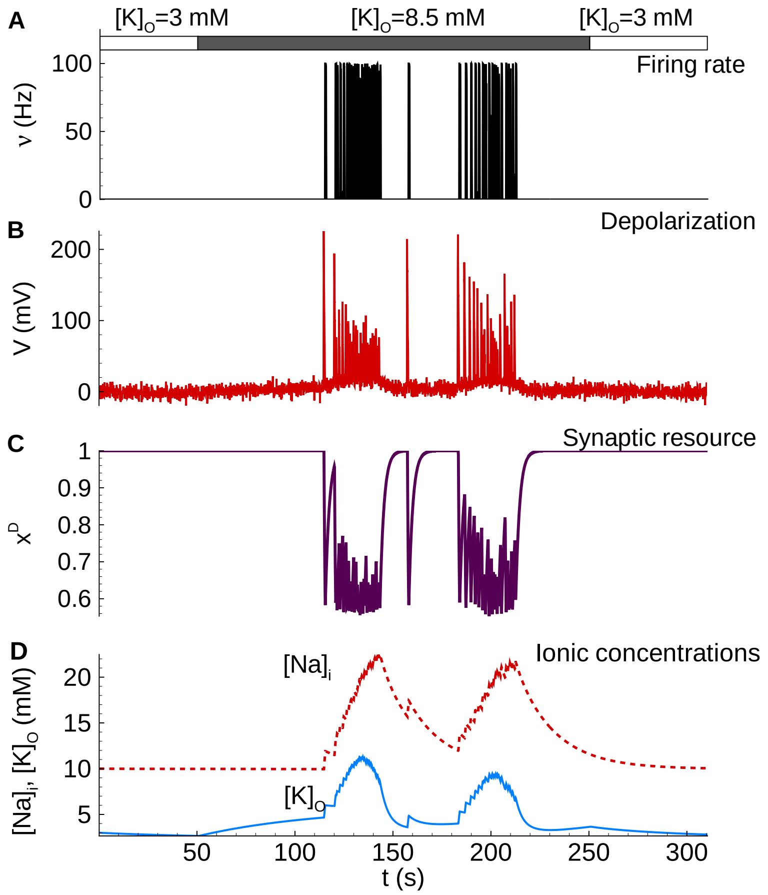

Fig. E. The transition from the resting state at $[K]_{bath} = 3\,mM$ to the ID generation ($\tau_K = 100\,s$) at $[K]_{bath} = 8.5\,mM$ at the time moment $t = 50\,s$ and back at $t = 250\,s$. The legend is in Fig. 3 of the main text.

6. The fact that each SB increases $[K]_o$ is evident from Fig. 3F,G in the main text.
7. A relatively rapid increase of $[K]_o$ leads to SBs clustering. This fact is supported by the fact that relatively low variation of $[K]_o$ as in the case of more rapid synaptic recovery (Fig. 3 in the main text) does not result in significant clustering of SBs. On the other hand, if $[K]_o$ modulates significantly, it changes the frequency of SBs. The dependence of the SB frequency on $[K]_o$ is demonstrated in Fig. F.

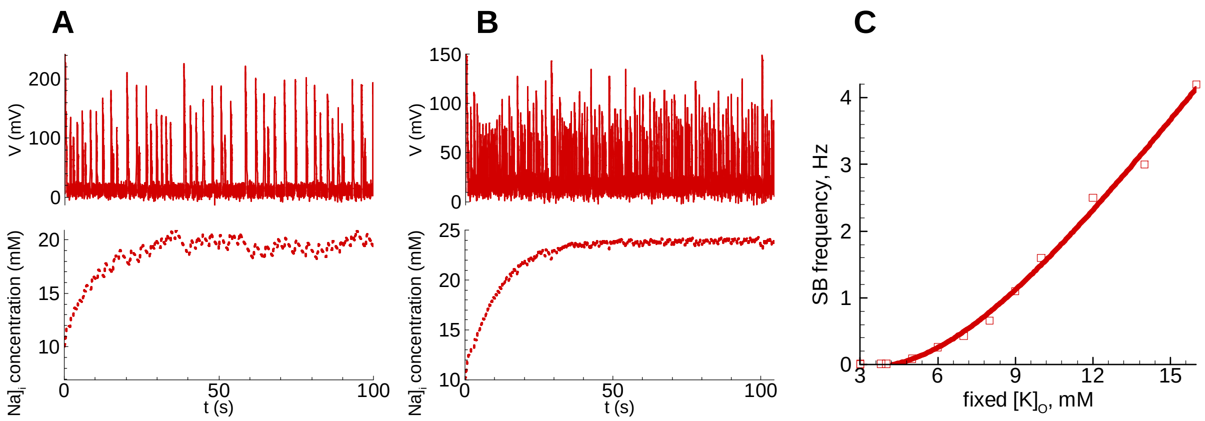

Fig. F. The dependence of the frequency of SBs on fixed $[K]_o$. **(A)** $[K]_o = 7\,mM$. **(B)** $[K]_o = 10\,mM$. **(C)** Cumulative result.

8. SBs clustering leads to the enhancement of $[Na]_i$. If the sodium accumulation at spikes is not so effective, then $[Na]_i$ does not reach that level of Na-K-pump activation that provides IDs. In this case, $[K]_o$ keeps growing, so the SB generation does not terminate. This is demonstrated in Fig. G.

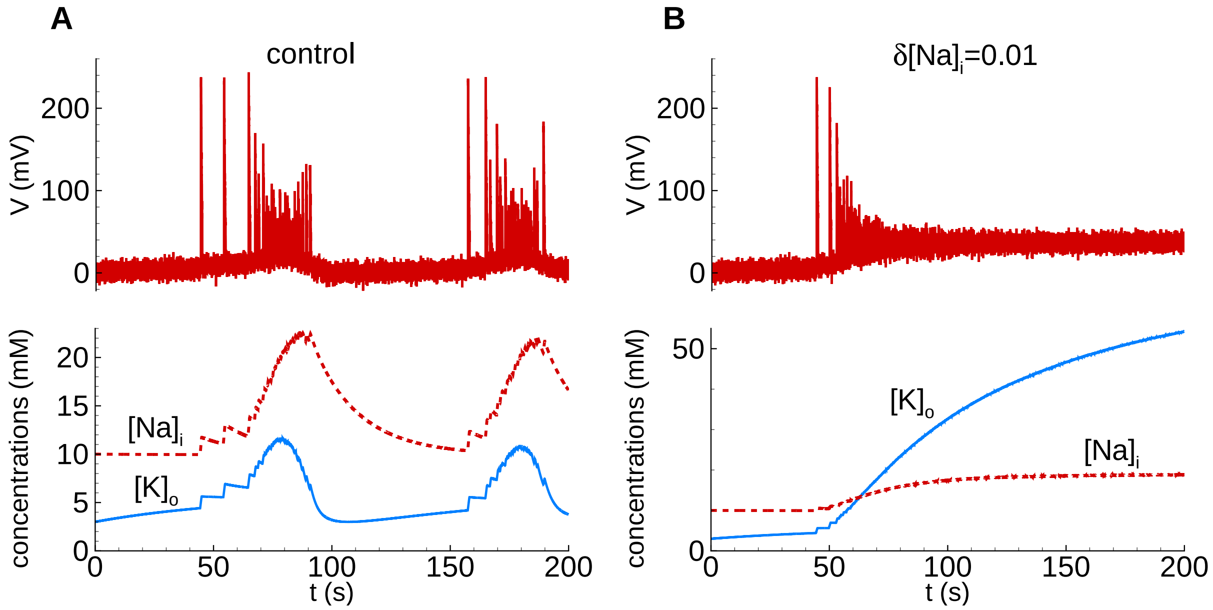

Fig. G. Decreased sodium increment $\delta[Na]_i$ results in a lack of IDs, because $[K]_o$ keeps growing.
(A) Control case with IDs. **(B)** $\delta[Na]_i = 0.01 mM/s$.

9. The enhanced $[Na]_i$ effectively activates the Na-K-pump. The nonlinear dependence of the Na-K-pump current on the concentrations $[K]_o$ and $[Na]_i$ is shown in Fig. H. As seen, at the moderate and high $[K]_o$ (above approximately 5 mM) the pump rate mostly depends on $[Na]_i$ and rapidly increases when $[Na]_i$ reaches about 20 mM.

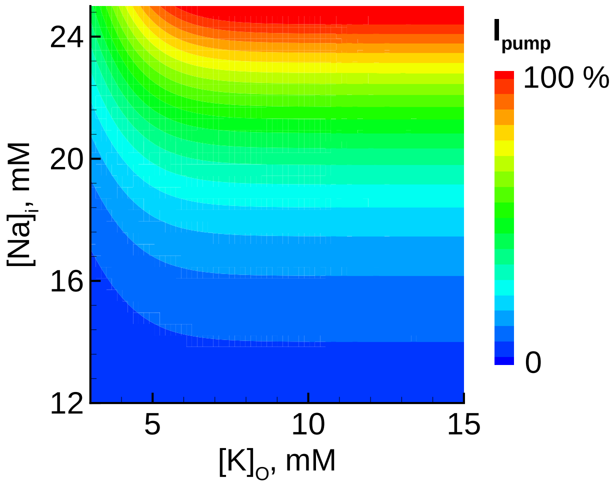

Fig. H. The dependence of the Na-K-pump rate on the concentrations $[K]_o$ and $[Na]_i$, according to eq.(8).

10. Na-K-pump decrease $[K]_o$. If the density of the Na-K-pumps is low, then the IDs might last longer or transfer to non-interruptive spiking regime (Fig. I).

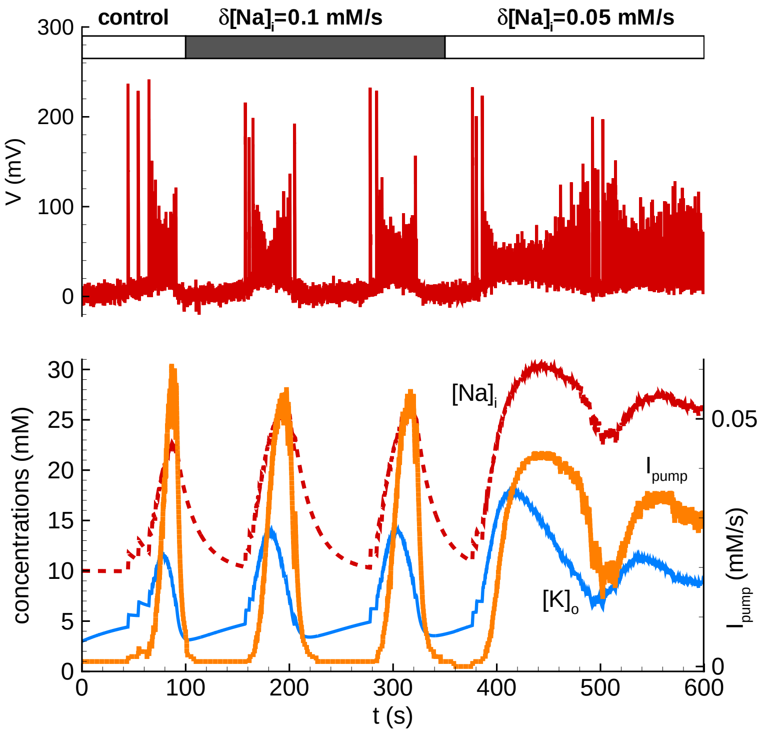

Fig. I. The dependence of the ID generation on the Na-K-pump rate on the density of Na-K-pumps.

11. The bath diffusion returns $[K]_o$ to the level of SBs generation. The smaller the diffusion coefficient, the bigger the interval between IDs, i.e., the interictal interval. In Fig. J the diffusion time constant τ_K was increased after the second ID, which resulted in the enlargement of the interval between the second, third and fourth IDs.

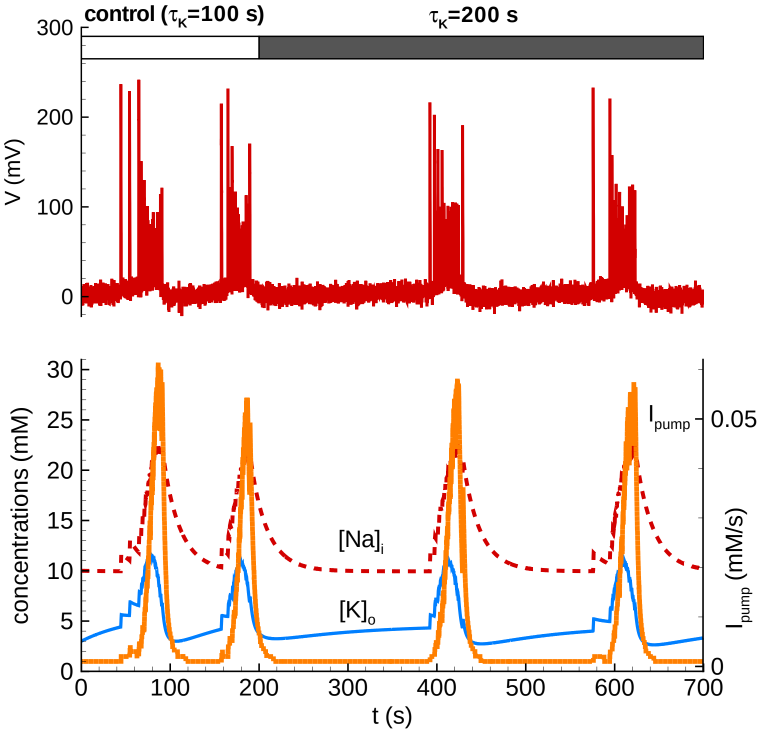

Fig. J. The dependence of the interictal interval on the Na-K-pump rate on the diffusion time constant τ_K.

Summarizing, all stages of the IID and ID generation mechanisms have been illustrated by the simulations described above. The roles of key factors have been clarified.

2. Disinhibition as a model of epilepsy.

Our model of epilepsy, considered in the paper, implies both mechanisms – disinhibition and an increase of extracellular potassium concentration by means of elevated potassium concentration in a bath. This model is close to the models that we used in our experiments (Amakhin et al. // Front. Cell. Neurosc. 2016; Chizhov et al. // PloS One 2017). An alternative model of epilepsy is based on disinhibition only. Below we consider a simulation with normal extracellular and bath potassium concentrations, 3 and 4 mM, correspondingly. The effect of partial disinhibition is modeled by changing the ratio of inhibitory versus excitatory synaptic currents. This ratio is given by the coefficient in eq.(6), which is fixed to be 0.5 in the simulations described in the main paper. Here, to model a normal level of inhibition, balanced with the excitation, we change the coefficient and set it to be equal to 1 for a "control" case. The partial disinhibition is modeled by setting the coefficient to 0.5. The simulations are shown in Fig. K. The system is silent in the control case and switches to ID generation in the case of partial disinhibition. The state is reversed by returning the control conditions.

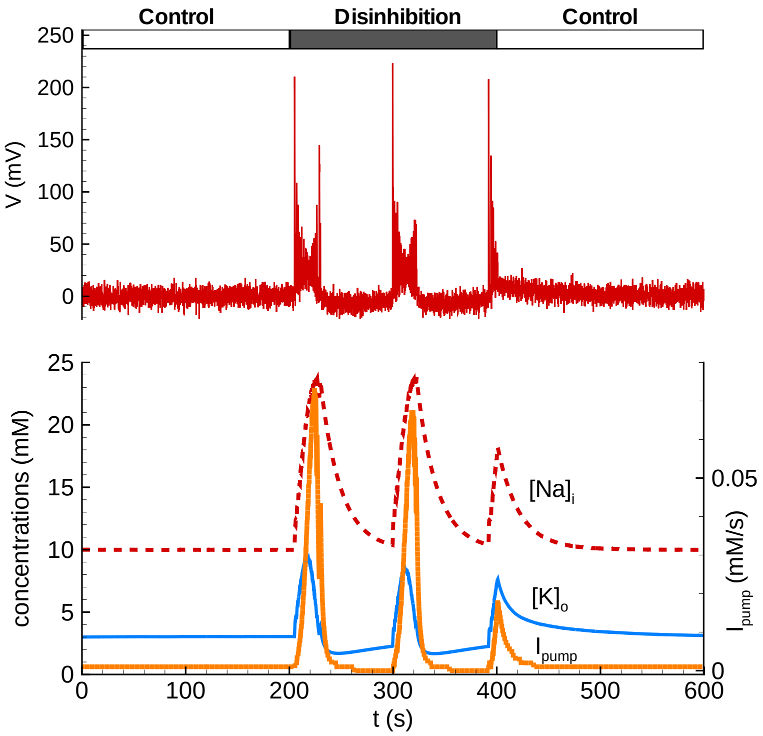

Fig. K. Disinhibition as a model of epilepsy. The control conditions are set by $V_{th} = 15\,mV$, coefficient 1 instead of 0.5 in eq.(6), and $[K]_{bath} = 4\,mM$. The partial disinhibition is set by the coefficient 0.5.

3. Depolarization block.

The depolarization block (DB) is sometimes observed in intracellular recordings during IDs, mainly in the interneurons. However, this factor has not been included in our Epileptor-2 model. The reasoning for this is following. First, the depolarization block is not a frequent effect in our recordings (Amakhin et al. // Front. Cell. Neurosc. 2016; Chizhov et al. // PloS One 2017). Second, the block of interneurons might lead to a temporal lack of inhibition during IDs, i.e., provides a pro-epileptic effect. At the same time, there are several pro-epileptic factors that support the ID generation, but a challenge to modeling is to reveal those factors that terminate each of IIDs and IDs. That is why, for the sake of simplicity, DB is excluded from the main model. However, in order to demonstrate its effect, here we modify eq.(6) in the following way.

$$u(t) = \begin{cases} u_1(t) + u_{ex}(t) + u_{in}(t), & if \quad u_1(t) + u_{ex}(t) + u_{in}(t) < u_{DB}; \\ u_1(t) + u_{ex}(t), & othewise \end{cases} \qquad (6')$$

with $u_1(t) = g_{K,leak}(V_K(t) - V_K^0) + \sigma\,\xi(t)$, $u_{ex}(t) = G_{syn}\,\nu(t)\,x^D(t)$, $u_{in}(t) = -0.5\,G_{syn}\,\nu(t)$. By this way, we imply that if the total current that drives interneurons exceeds the threshold u_{DB}, then the interneurons are blocked by the depolarization and do no longer contribute into the total current $u(t)$. The formula (6’) converges to (6) for infinitely large u_{DB}.

In simulations with eq. (6’) instead of (6) we set $u_{DB} = \infty$ for the conditions without DB and introduce it with $u_{DB} = 100\,mV$. The result is shown in Fig. L. As expected the depolarization was stronger with taking DB into account. The other characteristics of the IDs were rather the same. This simulation approves the neglect of DB in the Epileptor-2 model.

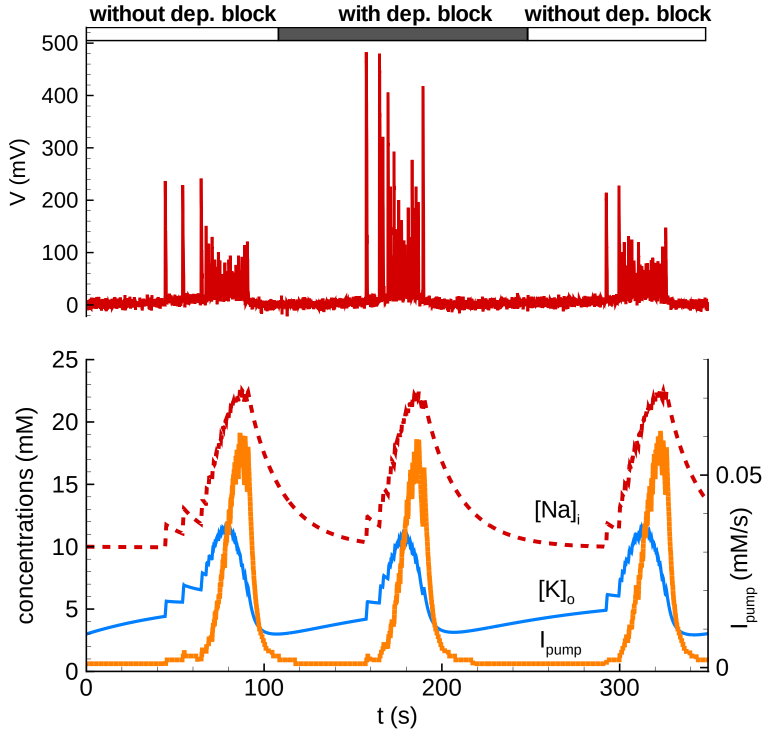

Fig. L. Role of the depolarization block in the ID generation.

4. An alternative model of a neuron-observer.

In the paper, we have described a quadratic integrate-and-fire (QIF) model as a model of neuron-observer. Here we compare QIF with adaptive QIF models (Fig. M).

The addition of a second variable, w, allows for the inclusion of an adaptation and captures the bursting behavior of neurons (Izhikevich // IEEE Transactions on Neural Networks 2003). The equations for the membrane potential $U(t)$ and the adaptation current $w(t)$ are as follows:

$$C\frac{dU}{dt} = g_U(U-U_1)(U-U_2) - w + u + I_a$$

$$\tau_w \frac{dw}{dt} = -w$$

$$if \quad U > V^T \quad then \quad U = V_{reset}, \quad w = w + \delta w$$

with the parameters close to those from (Zheng and Tonnelier 2009): $g_U = 1.5\,nS/mV$, $C = 1nF$, $\tau_w = 200\,ms$, $V^T = 25\,mV$, $V_{reset} = -40\,mV$, $\delta w = 100\,pA$, $U_1 = -60\,mV$, $U_2 = -40\,mV$; initial conditions are $U = -70\,mV$; $w = 0$; the tonic current is $I_a = 116\,pA$.

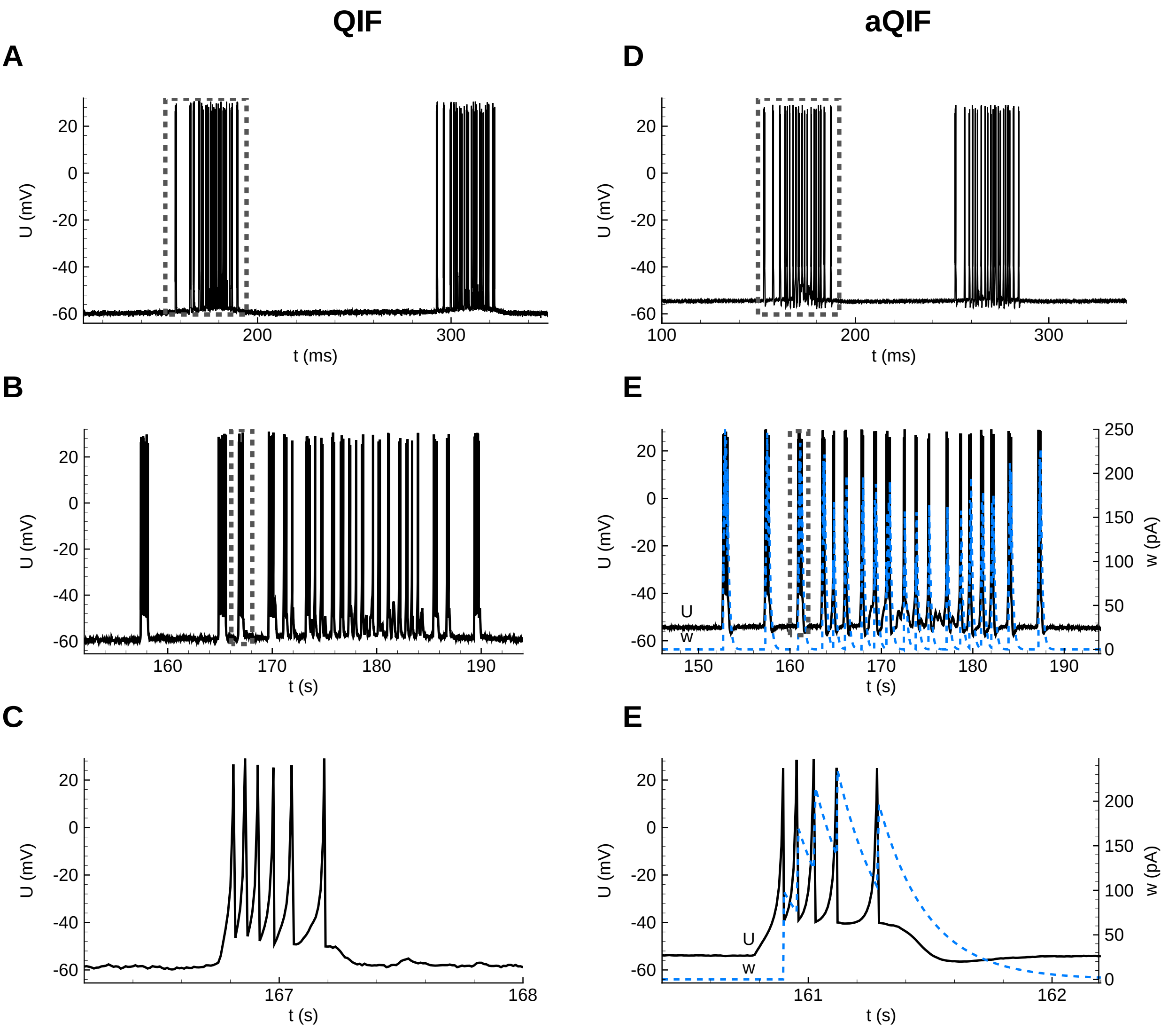

Fig. M. Comparison of QIF (A-C) and aQIF (D-F) models of a neuron-observer. Simulations of a single neuron activity during the IDs shown in Fig. 4 in the main text. **A,D**, two IDs as bursts of clustered SBs, seen in the membrane voltage. **B,E**, a single ID containing a number of SBs. Membrane voltage (black line, left axis) and the adaptation current (blue line, right axis). **C,F**, a single SB.

References

1. Zheng G, Tonnelier A. Chaotic solutions in the quadratic integrate-and-fire neuron with adaptation. Cogn Neurodyn. 2009; 3: 197–204.

2. Izhikevich EM. Simple Model of Spiking Neurons IEEE Transactions on Neural Networks. 2003; 14: 1569-1572.

3. Amakhin DV, Ergina JL, Chizhov AV, Zaitsev AV. Synaptic Conductances during Interictal Discharges in Pyramidal Neurons of Rat Entorhinal Cortex. Front Cell Neurosci. 2016; 10: 233.
4. Chizhov A, Amakhin D, Zaitsev A. Computational model of interictal discharges triggered by interneurons. PLoS ONE. 2017; 12(10): e0185752.
